# Bayesian spatial modelling of early childhood development in Australian regions

**DOI:** 10.1186/s12942-020-00237-x

**Published:** 2020-10-19

**Authors:** Mu Li, Bernard Baffour, Alice Richardson

**Affiliations:** 1 Research School of Finance, Actuarial Studies and Statistics, Australian National University, Canberra, 2601 Australia; 2 School of Demography, Australian National University, Canberra, 2601 Australia; 3 Statistical Consulting Unit, Australian National University, Canberra, 2601 Australia

**Keywords:** Australian Early Development Census (AEDC), Developmental vulnerability, Bayesian modelling, Spatial smoothing, Socio-economic index

## Abstract

**Background:**

Children’s early development plays a vital role for maintaining healthy lives and influences future outcomes. It is also heavily affected by community factors which vary geographically. Direct methods do not provide a comprehensive picture of this variation, especially for areas with sparse populations and low data coverage. In the context of Australia, the Australian Early Development Census (AEDC) provides a measure of early child development upon school entry. There are two primary aims of this study: (i) provide improved prevalence estimates of children who are considered as developmentally vulnerable in regions across Australia; (ii) ascertain how social-economic disadvantage partly explains the spatial variation.

**Methods:**

We used Bayesian spatial hierarchical models with the Socio-economic Indexes for Areas (SEIFA) as a covariate to provide improved estimates of all 335 SA3 regions in Australia. The study included 308,953 children involved in the 2018 AEDC where 21.7% of them were considered to be developmentally vulnerable in at least one domain. There are five domains of developmental vulnerability—physical health and wellbeing; social competence; emotional maturity; language and cognitive skills; and communication and general knowledge.

**Results:**

There are significant improvements in estimation of the prevalence of developmental vulnerability through incorporating the socio-economic disadvantage in an area. These improvements persist in all five domains—the largest improvements occurred in the Language and Cognitive Skills domain. In addition, our results reveal that there is an important geographical dimension to developmental vulnerability in Australia.

**Conclusion:**

Sparsely populated areas in sample surveys lead to unreliable direct estimates of the relatively small prevalence of child vulnerability. Bayesian spatial modelling can account for the spatial patterns in childhood vulnerability while including the impact of socio-economic disadvantage on geographic variation. Further investigation, using a broader range of covariates, could shed more light on explaining this spatial variation.

## Introduction

Early childhood development is a critically important part of achieving good health outcomes for children through to adulthood. Furthermore, like most phenomena, the impact of early childhood development on future health and social outcomes is influenced by community factors where a person lives [1–3]. While the evidence for geographic variation in early childhood development is strong [4–7], comprehensive data is not always available to describe this variation well, particularly in areas where populations may be sparse and surveys may have incomplete coverage. As a result, most analyses have been based on relatively broad geographic areas in order to have large enough samples, particularly when considering outcomes that are rare and affect a small sub-section of the population.

In an Australian context, information on child development has been collected regularly through the Australian Early Development Census as part of the government’s commitment to measuring the health and wellbeing of children [8]. While the majority of children are thriving and developmentally on track, one in ten children are considered to be developmentally vulnerable nationally. However, at a subnational level there are differences in the developmental vulnerability, and stark differences are exhibited at lower levels of geography. This observed geographical difference is due to the fact that vulnerable child development is not relatively common in the population, and as such the sample sizes are usually not large enough to provide reliable estimates for disaggregated analysis.

Often, maps are used to explore the relationship between developmental indicators and neighbourhood characteristics, and can be used to identify locations for targeted intervention [9, 10]. However, mapping of direct estimates of developmental vulnerability at high degrees of resolution using information only from fully observed regions is problematic for several reasons. Firstly, the estimates may be based on small samples and so have high variability. Secondly, there may be no sample data in certain small areas leading to blank spaces on the map. Thirdly, there is no account taken of the spatial correlation between small areas, so that estimates on adjacent small areas may vary a lot due mostly to sampling variability, leading to misleading appearance of statistically significant local high rate clusters (‘hot spots’) or low rate clusters (‘cold spots’) in a map. These issues are international in scope as Australia is one of many countries with a large variation in population density across the country.

Further, there is an expansive literature that has examined the impact of poverty (i.e. socio-economic disadvantage) on early childhood development [11–13], and whether you are poor is related to where you live [14–17]. In this paper we provide improved small area estimates of the prevalence of developmentally vulnerable children and assess the extent to which socio-economic disadvantage could explain the observed geographical and spatial variation. We illustrate this through modelling the proportion of developmentally vulnerable pre-school children at a high level of geographic resolution in Australia, whilst accounting for both the relative disadvantage and spatial relationship between small areas. We find significant improvements in the precision and reliability of the estimates of the prevalence of developmental vulnerability.

There have been a number of interventions launched in Australia to improve the psychosocial conditions linked to early childhood development (for example, the Support at Home for Early Language and Literacies (SHELLS) [18] and Triple P—Positive Parenting Program [19]). However, owing to the geographical distribution of Australia with a large urbanised population who live along the coastline–80% of the population live in the narrow strip of land between Adelaide and Brisbane, along the coast on an area roughly 3% of the country’s land mass [20], the evaluation of these programs at a national level is fraught with difficulty [21]. Moreover, there are regional variations in vulnerability due to population distribution and the fact that rural and remote areas tend to be more socially deprived [22].

## Data and methods

### Data

#### AEDC

Since 2009, and conducted every 3 years, the Australian Early Development Census (AEDC) has been used to collect data across public, private and independent schools in the entire country and provides a population-based measure of children’s development as they enter their first year of full-time school [23, 24]. The instrument used to measure child development has been adapted from the Canadian Early Development Instrument [25], demonstrating international applicability of the methods in this paper. Teachers complete the instrument for all first-year school children (generally aged 5 years old). The responses provide information on the five domains of children development that the data is used to measure—physical health and wellbeing, social competence, emotional maturity, language and cognitive development, and communication skills. These domains of development are considered to provide a snapshot of a child’s level of school readiness, which is an important predictor of ongoing educational and occupational achievement and general knowledge [26]. Data is collected for individual children and then reported for a group of children at a community, state/territory, or national level [5, 27]. For each domain, children are given scores based on their teachers’ responses to questionnaire and these scores are used to assess the extent of their development. For each of the five AEDC domains, children receive a score between zero and ten, where zero is most developmentally vulnerable. AEDC results are reported as the percentage of children who are considered to be ‘developmentally on track’, ‘developmentally at risk’ and ‘developmentally vulnerable’ on each domain.

Children are categorized as ‘developmentally on track’, ‘developmentally at risk’, or ‘developmentally vulnerable’, based on a series of cut-off scores. To create these cut-offs, all children’s domain scores were sorted and ranked from lowest to highest. Scores ranked in the lowest 10% were classified as ‘developmentally vulnerable’; scores between 10% and 25% were classified as ‘developmentally at risk’; the remaining scores (ranked in the highest 75%) were classified as ‘developmentally on track’. The cut-off scores used in the first cycle in 2009 have remained the same across each subsequent collection cycle to provide a reference point against which later AEDC results can be compared [27, 28].

The participation rate for the first cycle of the AEDC in 2009 was 97.5% of eligible children. The second cycle of the AEDC in 2012 collected information on 96.5% of all Australian children registered to commence school in 2012. The overall participation rate in 2015 (third cycle) was also 96.5% of all registered children, and the most recent data collection in 2018 (fourth cycle) achieved an almost identical participation rate of 96.4% [23]. Microdata access to the unit record data is restricted, for confidentiality and privacy concerns, and for this reason publicly available AEDC aggregate data is provided at two geographies called ‘communities’ or ‘local communities’. These geographies are defined for the whole country to ensure that the data is reported in the most useful way possible, yet still align with commonly understood geography, such as suburbs and administrative regions. Accordingly, AEDC communities represent Local Government Areas, while AEDC local communities represent suburbs.

However, since Australia has unique settlement patterns—with both densely populated urban areas and sparsely populated remote areas—there is remarkable variation in terms of the local community geography. These local community geographies have a positively skewed population distribution, with many smaller communities which can also be spread over a large number of square kilometres. The distribution of the number of children in an AEDC local community can range from one child at one end of the spectrum to 816 children at the other end, although the mean number of children in a community is 64, and the median is 41. Further, while the mean community population size is 4216 (with the median size of 2701 people), there are some inner metropolitan areas with populations of over 45,000 and in metropolitan and large regional areas the mean population size is roughly 10,000 people [27]. In the fourth cycle (in 2018), the AEDC geography was updated in order to align with the new Australian Statistical Geography Standard (ASGS) released by the Australian Bureau of Statistics (ABS) [29]. The geographical boundaries community and local communities have been matched and then updated in order to harmonize the data over all four cycles (from 2009 to 2018). This update to align AEDC geographies to ABS standard geographies (i.e. statistical areas) allows the pairing of the AEDC data to the Socioeconomic Index for Areas (SEIFA) produced by the ABS after each census.

#### SEIFA

The Socio-economic Indexes for Areas (SEIFA) is a summary measure of the socio-economic condition of a specific local area. The SEIFA are calculated using principal components analysis of several variables—demographics (e.g. gender-age distribution,  % of single parents with dependent children and  % lacking fluency in English), income (e.g.  % in lowest household equivalised income bracket), employment and education (% unemployed (in labour force) and  % with university qualification) and housing and health (% owners,  % households with no Internet connection and  % with a long-term limiting health condition or disability) [30].

SEIFA comprises four indexes: Index of Relative Socio-Economic Disadvantage (IRSD); Index of Relative Socio-Economic Advantage and Disadvantage (IRSAD); Index of Education and Occupation (IEO); and The Index of Economic Resources (IER).

For our work, since the literature on neighbourhood effects suggests that it is relative disadvantage that affects child outcomes, rather than advantage acting as a protective factor [31, 32], we used the Index of Relative Socio-Economic Disadvantage (IRSD). The SEIFA-IRSD ranks areas on a continuum from most disadvantaged to least disadvantaged. A low score on this index indicates a high proportion of relatively disadvantaged people in an area. The converse is not true: an area with a high score does not mean that it has a high proportion of relatively advantaged people, but that the area has a relatively low incidence of disadvantage [33].

We use IRSD as an auxiliary covariate since it has been well-established that the socio-economic condition of an area is highly correlated with child development [7, 34]. The covariate effects account for the spatial patterns due to areas that are socio-economically similar tending to have similar values of child vulnerability and can be used to borrow strength over neighbouring regions to obtain more reliable region-specific estimates. In the Bayesian model we combine the prior knowledge about the intrinsic behaviour of spatially related data, and this spatial structure is specified with a set of spatially autocorrelated random effects, in addition to the potentially available covariate information.

#### Geographical unit of analysis and analytic sample

We focus on the third level of Australian statistical geography (SA3) as they are designed for output of regional data, and are formed by clustering groups of areas that have similar regional characteristics, administrative boundaries or labour markets. They are often functional areas of regional towns and cities with a population in excess of 20,000 or clusters of related suburbs around urban commercial and transport hubs within the major urban areas. Generally, SA3s have populations of between 30,000 and 130,000 [29].

We use the SA3 as our geographical unit of analysis for two reasons. Firstly, the measurement of vulnerable child development as a general phenomenon is a relatively low frequency occurrence in the Australian population [5, 27]. As such, to accurately measure occurrence in an area, reasonably large sample sizes are needed. Secondly, due to the population distribution and settlement patterns in Australia, this regional level of analysis provides valuable insights into the spatial diversity of early childhood developmental vulnerability in Australia. Australia is comprised of 358 contiguous SA3 regions. However, this includes 18 areas with no resident populations (comprised of ‘Migratory-Offshore-Shipping’, and ‘no usual address’ codes for each State and Territory). Further there are 5 ‘island’ locations of Cocos (Keeling) Islands, Christmas Island, Norfolk Island, Lord Howe Island, and Jervis Bay.

These 23 isolated SA3 regions were removed since our spatial analysis requires pairwise neighbouring regions to compute an adjacency matrix and our final analytic sample used 335 SA3 spatial regions. The SEIFA data is usually downloaded at a sub-regional level (SA2) which broadly represents communities that interact socially and economically and similar in size to suburbs and are called by suburb names. There are roughly 2148 SA2s covering Australia (excluding ‘island’ and non-resident areas). To create the SA3 SEIFA index score, the population-weighted averages of the sub-regional (SA2) scores within each of the regions (SA3s) were calculated [33]. Two SA3 regions (Blue Mountains South and Illawarra Catchment, both in New South Wales) are very sparsely populated with less than 10 people and therefore it was not possible to calculate the SEIFA scores. These regions are included in our analytical sample as ‘missing auxiliary covariates’. In addition, there are two SA3 regions that have no valid responses (but the total sample size in nonzero) in the AEDC data. These regions, located in the Australian Capital Territory, are Canberra East and Urriarra—Namadgi, and have ‘missing responses’. In fact, for both these regions, the data have been suppressed (for confidentiality purposes) due to having too few observations: a criterion for reporting AEDC data includes a minimum of 15 children [27]. While in both instances these data are missing, the mechanisms under which the data are missing are different, and as such will be dealt with in a subsequent section.

### Methods

#### Spatial smoothing

As mentioned earlier, there is significant geographical variation in child development across Australia. Furthermore, the influences of socio-economic disadvantage on child vulnerability differ by geography, and only through properly taking into account geography can the spatial patterns underlying the phenomenon be identified [35]. When analysing spatial data, it is important to account for both spatial autocorrelation and sampling variability. Spatial autocorrelation refers to the idea that observations taken at locations near to each other tend to be similar [35–37], while sampling variability refers to differences between areas due to small populations or heterogeneity of individuals within areas [38–40], and in our circumstances it captures the heterogeneity due to differences in completeness of school census registers at a local level. Spatial smoothing firstly can remediate sampling variability through borrowing information from neighbouring areas, and effectively estimating the underlying rate; rather than simply reporting the observed data, which may susceptible to random variation due to sparse data when there are few reported observations. Secondly, spatial smoothing alleviates the effect of the sometimes arbitrary nature of boundaries defining geographical areas [41, 42].

There have been numerous statistical models which have been developed to address these issues of spatial data. Standard regression approaches which model the spatial structure by known explanatory variables (such as demographic attributes, social determinants, and covariate risk factors) can explain a proportion of this spatial autocorrelation. However, even after controlling for these covariate effects, there is significant residual spatial autocorrelation due to unmeasured confounding, neighbourhood effects and grouping effects [43].

The most common way of carrying out statistical spatial smoothing is to specifically include terms to account for spatial autocorrelation and sampling variability so as to satisfy model assumptions and reduce uncertainty of the estimates. This approach models the observed data using a Bayesian generalized linear mixed model (GLMM) [44, 45] to augment the linear predictor with a set of spatially autocorrelated random effects. This spatial correlation between areas is readily incorporated through the prior information, and recognizing that neighbouring geographical areas are more likely to share similar characteristics (i.e. through spatial dependence). This is known as the adjacency structure of areal units and the conditional autoregressive (CAR) model has been shown to induce this type of spatial autocorrelation [46–48]. Essentially, the model includes an additional unstructured spatial random effect term to account for the independent region-specific noise.

#### Spatial weights

An important feature of spatial models is the need to specify the spatial adjacency (neighbourhood) matrix. This matrix basically describes the spatial proximity between random effects for each pair of areas. This usually takes the form of a spatial weight matrix, $W$ [46]. There are several ways to define spatial proximity, which can be either continuous, for example, the centroid distance between areas, or discrete, for example, belonging to the neighbourhood of adjacent areas. The most common definition is the binary, first order, adjacency weights matrix [42], which takes the form $w_{ij} = 1$ if areas $i$ and $j$ are adjacent to each other; $w_{ij} = 0$ if otherwise.

In our model, we use this basic binary adjacency spatial weight. Since, there are a number of areas with multiple neighbours, we use the ‘Queen’ coding criterion [49], similar to the Queen definition in chess which means that all regions sharing at least one border would be defined as the neighbours and the corresponding element in the adjacency matrix is coded as 1. The corresponding entries of all regions that do not share any border will be zero, and the diagonal elements are all zero. More complex specifications of the adjacency matrix, for instance using a distance-based measure introduce model complexity, unrealistic spatial dependence, and do not necessarily lead to better inference [50, 51].

#### Bayesian spatial models

The modelling approach we employ uses a three-stage Bayesian hierarchical model to identify a smooth pattern in the outcome that may be explained using underlying covariates and spatial factors [41, 52] following the standard framework described in [53]. At the first stage, the likelihood for the data is specified by some distribution belonging to the exponential family; in the second stage, the expectation of the response variable is related to the linear predictor through a link function; and at the third stage the parameters in the linear predictor are assigned prior distributions. The use of (conditional autoregressive) prior distributions to specify the unknown parameters is especially helpful for quantifying the spatial parameters, since they can be used to impose structure on the underlying random process to ensure the closer areas are, the more related they are. The resulting spatial smoothing, or shrinkage, pulls the posterior estimates towards either a ‘global’ average (where there is a common spatial smoothing term across the region), or a ‘local’ average (which allows for differential smoothing depending on neighbourhood characteristics). This has the benefit of improving the stability of the estimates, and hence provides more robust estimates, especially for areas with sparse populations [46]. The use of a Bayesian modelling framework also means that the estimation borrows strength from both the data from the neighbouring observations and the auxiliary data about the neighbourhood characteristics.

For our problem, firstly, we specify that the outcome, the number of developmentally vulnerable children in an area, follows a binomial distribution. At the second stage, the log risk of child vulnerability is linked to the spatially structured and unstructured components as well as any potential covariates. The remaining stage focuses on the priors. The so-called Besag-York-Mollie (BYM) model [47, 54] has been extensively used in modelling areal count data of rare diseases. Waller and Carlin [55] and Lee [56] have provided an overview and comparison of the BYM model and other Bayesian spatial models. However, the BYM model may lead to inaccurate results, especially when there is no spatial correlation in the data. Furthermore, the BYM model is faced with inherent issues (specifically in modelling epidemiological data): the population size in small areas must be known, and the spatially structured component is assumed to be independent of the unstructured component. For this reason, we settle on an extension of the BYM model called the Leroux model [48] since it accounts for the unstructured variability in space, and has been favoured in modelling Australian data [41, 42].

We use the CarBayes package [43] in R 3.6.3 [57] to implement the spatial generalised linear mixed modelling for areal data, with inference in a Bayesian setting using Markov Chain Monte Carlo (MCMC) simulation [58].

#### Model specification

For notational purposes, let us assume that our population U of size N consists of R non-overlapping and mutually exclusive small areas (or local regional areas). We use a subscript r to index the quantities belonging to local regional area $r \left( {r = 1, \ldots , R} \right)$. Let $U_{r}$ and $N_{r}$ be population and size, respectively, in the $r$ th local regional area respectively such that $U\;\text{ = }\; \cup_{{r\text{ = }1}}^{R} \;U_{r}$ and $N\;\text{ = }\;\sum\limits_{{r\text{ = }1}}^{R} {N_{r} }$.

In addition, let $y_{rk}$ denote the value of a binary variable of interest for unit k in region r. With this, our aim is to estimate the small area population counts $y_{r} \;\text{ = }\;\sum\nolimits_{{k \in U_{r} }} {y_{rk} }$, i.e. the total number of developmentally vulnerable children in region ${\text{r}}$ or equivalently the small area proportions $p_{r} = N_{r}^{ - 1} \left( {\sum\nolimits_{{k \in U_{r} }} {y_{rk} } } \right)$.

Specified this way, it is reasonable to assume that the response $y_{r}$ follows a binomial distribution with parameters, $N_{r}$ and $p_{r}$, such that $y_{r} \sim Binomial\left( {N_{r} ,p_{r} } \right)$, where $p_{r}$ is the probability of being a developmentally vulnerable child in region $r$. Conversely, it follows that $\left( {1 - p_{r} } \right)$ is the probability of not being developmentally vulnerable. We refer to this probability as the prevalence of child developmental vulnerability. Note that the binomial distribution is reasonable under simple random sampling with replacement within each region. In addition, the binomial distribution will cope better with overdispersion for count data for small area spatial data [59].

We assume that the counts $y_{r}$ together with a p-vector of area-specific covariates ${\text{X}}_{r}$ derived from secondary data sources are available, for each of 335 SA3 regions. The model linking the probability of being developmentally vulnerable (i.e. the vulnerability prevalence) with the explanatory covariates is the logistic linear mixed model of form

1 $$logit\left( {p_{r} } \right) = log\left\{ {\frac{{p_{r} }}{{1 - p_{r} }}} \right\} =\varvec{\eta}_{r} = \log \left\{ {{\mathbf{e}}_{\text{r}} } \right\} + \theta_{r}$$

2 $$\theta_{r} = \alpha + \varvec{X}_{r}^{T}\varvec{\beta}+ \varvec{u}_{r} + \varvec{v}_{r} , r = 1, 2, \ldots , 335.$$

Here $\varvec{e}_{\varvec{r}}$ is the expected number of developmentally vulnerable children in region r; $\theta_{r}$ is the log relative risk of being a developmentally vulnerable child; $\alpha$ is the overall level of relative risk; $\varvec{\beta}$ is the p-vector of regression coefficient often known as fixed effect parameters; $\varvec{u}_{r}$ is the region-specific spatially structured random effect that accounts for between regions dissimilarity beyond that explained by the auxiliary variables included in the fixed part of the model; and $\varvec{v}_{r}$ is the remaining random effect which is purely overdispersion.

We assume that this effect has a conditionally autoregressive (CAR) prior structure. Following, Besag, York and Mollie [47] and Leroux et al. [48], amongst others, we use an intrinsic Gaussian conditionally autoregressive (ICAR) prior model, where the areas $i$ and $j$ are neighbours if they both share a common border, denoted here as $i\sim j$. For CAR models, the neighbour relationship is symmetric, however, it is not reflexive, and this means that if $i\sim j$ then $j\sim i$, but, a region is not its own neighbour. As such a binary neighbourhood weighting spatial parameter is specified as

3 $$\left( {u_{j} |u_{i} , j \ne i, \tau_{u}^{2} } \right)\sim N\left( {\frac{{\mathop \sum \nolimits_{i} u_{i} w_{ji} }}{{\mathop \sum \nolimits_{i} w_{ji} }}, \frac{{\tau_{u}^{2} }}{{\mathop \sum \nolimits_{i} w_{ji} }}} \right)$$

where $w_{ji} = 1$ if $i$ and $j$ are adjacent, and $w_{ji} = 0$, otherwise [46–48, 54]. Finally, we also assume, $\varvec{v}_{r}$ is a spatially unstructured random effect with mean zero and variance $\tau_{v}^{2}$. This model decomposes the total random variation into region-specific structured and unstructured components. The structured spatial effects of a particular region depend on the effects of neighbouring regions, while the unstructured spatial random effects account for independent region-specific noise.

#### Specification of hyper priors of the variance terms

Under the Bayesian setting using MCMC simulation, the extent of smoothing depends on both the data and specific prior distribution used, and proper specification of the priors will lead to greater computational efficiency [60]. In the original BYM model formulation, priors are usually specified separately for $\tau_{u}^{2}$ and $\tau_{v}^{2}$, the structured and unstructured variance terms, respectively, but these priors are often selected on ad-hoc basis [61]. A better alternative is to express prior beliefs in terms of the total variability of the model [48] which also facilitates a clearer interpretation of the hyperparameters [62]. This model is more efficient to parameterize and solves the issues of model identifiability in decomposing the total random variation into structured and unstructured components [43]. Here, we apply weakly specified hyperpriors following a normal and inverse gamma distribution, respectively, and our final choice made based on goodness of fit, computation time and plausibility of estimates [50].

## Analysis of spatial variation in prevalence of developmental vulnerability

### Descriptive statistics

In the AEDC data, the children have their respective scores in each domain that assesses the extent of their development. In the first data collection cycle in 2009, the children whose score are below the tenth percentile were categorised as ‘developmentally vulnerable’. In the subsequent data collection cycles (of 2012, 2015 and 2018), the 2009 ‘cut-off’ points for developmental vulnerability were used to ensure comparability over time [27, 28]. Our focus is on the last data collection cycle of 2018, and as our results (in Table 1) show there has been a decline in developmental vulnerability, and this holds for all five domains, compared to the baseline cycle in 2009, with 10% of developmentally vulnerable children in all the five domains. Over the whole of Australia, 9.6% of children are developmentally vulnerable according to the Physical and Wellbeing domain, 9.8% in Social Competence, 8.4% in Emotional Maturity, 6.6% in Language and Cognitive Skills, and 8.2% in Communication and General Knowledge. The region-specific crude prevalence rates for all the five early childhood development domains data are provided in the supplementary material.

**Table 1:** Proportion of Developmentally Vulnerable Children in the different Early Development Domains (with standard errors)

	Physical health and wellbeing (%)	Social competence (%)	Emotional maturity (%)	Language and cognitive skills (%)	Communication and general knowledge (%)
Overall (n = 335, m = 4)	9.6 (0.24)	9.8 (0.22)	8.4 (0.18)	6.6 (0.30)	8.2 (0.25)
*By State/Territory*					
New South Wales (n = 91, m = 2)	8.5 (0.32)	9.2 (0.30)	6.8 (0.22)	5.2 (0.29)	8.0 (0.33)
Victoria (n = 66)	8.2 (0.37)	8.8 (0.33)	8.1 (0.29)	6.4 (0.35)	7.4 (0.36)
Queensland (n = 82)	12.3 (0.43)	11.9 (0.35)	10.5 (0.31)	8.0 (0.41)	10.1 (0.40)
Western Australia (n = 34)	8.9 (0.56)	7.4 (0.57)	7.7 (0.45)	6.6 (0.77)	7.0 (0.50)
South Australia (n = 28)	10.8 (0.74)	11.5 (0.75)	10.8 (0.66)	7.2 (0.72)	8.4 (0.60)
Tasmania (n = 15)	9.5 (0.95)	8.8 (0.64)	9.2 (0.76)	8.0 (0.64)	5.7 (0.64)
Australian Capital Territory (n = 10, m = 2)	12.1 (0.93)	12.3 (1.29)	9.9 (0.98)	6.4 (0.63)	7.8 (0.79)
Northern Territory (n = 9)	17.6 (3.18)	17.8 (3.24)	14.9 (2.24)	19.6 (5.92)	16.7 (4.35)

n is the number of SA3s in each of states/territories

m is the number of SA3s that have non-valid data in each of the states/territories, and there are 4 SA3s with non-valid data (2 in New South Wales and 2 in the Australian Capital Territory)

We find variability in the prevalence of child developmental vulnerability in Australia using the direct data. As a reflection of the geographical distribution of the population in Australia, there is also spatial variation in the prevalence of child vulnerability in the different states/territories—this is exemplified by larger standard errors in the estimates in the smaller, sparsely populated, states and territories (particularly in the Northern Territory). Further, the distribution of the number of children in each region is also highly variable (see Table 2). Due to the inherent variability of the direct estimates as a result of variation both within and between regions, it is not advisable to inspect the crude prevalence rates directly. However, through spatial modelling we can borrow strength over neighbouring regions to get more reliable region-specific estimates.

**Table 2:** Summary of number of children participating in AEDC 2018 in each geographical region (SA3)

State	Number of SA3 regions	Mean number of children (standard error)	Minimum/Maximum	Interquartile range
Overall	335	878.2 (34.90)	0/4441	679
By State/Territory				
New South Wales	91	1027.7 (65.25)	0/2479	939
Victoria	66	1089.3 (102.96)	114/4441	981
Queensland	82	754.2 (51.06)	129/2502	398
South Australia	28	685.1 (89.55)	97/2048	473
Western Australia	34	966.6 (114.17)	119/3008	800
Tasmania	15	389.8 (62.83)	42/947	333
Australian Capital Territory	10	548.4 (164.14)	5/1335	849
Northern Territory	9	396.9 (68.81)	68/738	238

The Interquartile range is calculated as the difference between the upper quartile and the lower quartile (Q3–Q1)

### Model fitting

Our empirical strategy to find the best model which fully incorporates the socioeconomic and geographic influences on the proportion of developmentally vulnerable children in a region. To do this, we fit a regression model which firstly accounts for the spatial patterns underlying childhood vulnerability through fixed and random effects, and secondly borrows strength from neighbouring regions through spatial smoothing random effects to improve the final estimates of childhood vulnerability, especially in sparsely populated areas. Following on from previous studies [14, 63] we use a single auxiliary covariate based on the composite SEIFA measure (here the Index of Relative Socio-Economic Disadvantage) because it has the advantage of improving the efficiency of the model shown in Eq. (1) as it is parsimonious and reduces the potential number of covariates, which in turn minimises the regression model variability.

Our model is a special case of the generalised linear mixed model with logit link function adapted to the specific context of small area estimation. Under this model $p_{r} = p_{r} \left( {\beta , u_{r} , v_{r} } \right) = \frac{{{ \exp }\left( {X_{r}^{T} \beta + u_{r} + v_{r} } \right)}}{{1 + { \exp }\left( {X_{r}^{T} \beta + u_{r} + v_{r} } \right)}}$ and $E[y_{r} |u_{r} , v_{r} ]= N_{r} p_{r}.$ Estimation of the fixed effects parameters and the area-specific random effects uses data from all geographic regions. Also, the estimate of the prevalence of developmentally vulnerable children in a region, $r$ is given by $\hat{p}_{r\;} \text{ = }\;p_{r} \;\left( {\hat{\beta }\text{,}\;\hat{u}{}_{r}\text{,}\;\hat{v}_{r} } \right)\;\text{ = }\;\frac{{\exp \left( {X_{r}^{T} \hat{\beta }\text{,}\;\hat{u}{}_{r}\text{,}\;\hat{v}_{r} \;} \right)}}{{1\;\text{ + }\;\exp \left( {X_{r}^{T} \hat{\beta }\text{,}\;\hat{u}{}_{r}\text{,}\;\hat{v}_{r} } \right)}}$ . Here $\hat{\beta }\text{,}$ $\hat{u}_{r}$ and $\hat{v}_{r}$ are the estimate of the fixed effects parameter and the prediction of the structured and unstructured random effects parameter respectively under model (1). For inference on the precision parameters $\left( {\tau_{u} , \tau_{v} } \right)$ we set independent gamma priors such that, $\pi \left( {\tau_{u} |\alpha_{u} , \beta_{u} } \right) \propto \tau_{u}^{{\alpha_{u} - 1}} { \exp }\left( {\tau_{u} \beta_{u} } \right)$ and $\pi \left( {\tau_{v} |\alpha_{v} , \beta_{v} } \right) \propto \tau_{v}^{{\alpha_{v} - 1}} { \exp }\left( {\tau_{v} \beta_{v} } \right)$. We then choose $\alpha_{u} = \alpha_{v} = 1$, and $\beta_{u} = 0.5$ and $\beta_{v} = 0.01.$ The resulting posterior density can be integrated using MCMC sampling and approximation methods to estimate $\left( {\hat{\tau }_{u} \text{,}\;\hat{\tau }_{v} } \right)$.

### Dealing with missing data in spatial modelling

As described earlier our data contains 4 regional areas with non-valid observations of child vulnerability. These four areas have been suppressed for data confidentiality purposes. But there are two different manners in which the data are suppressed. On the one hand, there are two regions with no sample observations from the AEDC but there is census information available to compute SEIFA scores. We estimate the prevalence of child vulnerability (for each of the domains) through a plug-in estimator which uses the model estimated parameters and the SEIFA disadvantage score for the specified region (following a similar approach of out-of-sample prediction in Baffour et al. [14]). Under the mixed model (1), the predictor of the prevalence of child vulnerability for our out-of-sample areas is simply the average estimate $p_{r}\left( {\hat{\beta } , 0 , 0 } \right)$.

On the other hand, there are two regions with too few people in the geographical area, and as such do not have a SEIFA score. Here we used the Besag-York-Mollie (BYM) conditional autoregressive model with no auxiliary covariate to obtain predictions of the SEIFA score for those two areas from the smoothed data, following a similar approach taken in [64]. These estimates are subsequently plugged into model (1) to predict the prevalence of child vulnerability in these two areas with missing covariates.

### Model diagnostics

After the model-based estimation, it is important to determine whether the model assumptions are satisfied before undertaking any inferences, and we accomplished this through a series of diagnostic procedures. We firstly examined whether the assumptions of the underlying model-based estimates were met, that is, how well the working model performed when fitted to data. We checked the random effect terms to see if they followed a random normal distribution, with no patterned residuals through looking at the q–q plots. Secondly, we also examined the validity of derived model-based estimates. The model-based estimates should be consistent with the unbiased direct estimates, be more precise than the direct estimates and provide reasonable results to users. We also undertook a sensitivity analysis to examine the robustness of the results with respect to different priors for the unstructured random effects, and the results remained unchanged.

## Results

In this section we present some results on the performance of the estimation of prevalence of child developmental vulnerability in Australia, through comparing the direct and model-based estimates. The remaining results are provided in the Appendix. The small area estimates produced by the model-based approach are subject to uncertainty because of the sampling and modelling processes. It is important to assess whether the model-based estimates are not only consistent with the direct estimates, but are also more precise. We determine the precision and reliability of the estimates through the coefficient of variation (CV), which is computed $\frac{{\sigma \left( {\hat{p}_{r} } \right)}}{{\hat{p}_{r} }} \times \;100{\% }$, where $\hat{p}_{r}$ is the estimated prevalence in region $r$, and $\sigma \left( {\hat{p}_{r} } \right)$ is the standard deviation. The CV provides a measure of relative errors and gives an indication of the precision of the model-based estimates when contrasted with the direct estimates. We expect the model-based estimates to be less extreme, and therefore have a smaller range of CVs and thereby demonstrate that the typical small area estimation behaviour of shrinking more extreme values towards to average. To provide an indication of the improvement provided by the model-based small area estimation, we computed a measure based on the difference of the (percentage) CV between the direct and model-based estimate, $CV_{r}^{o} - CV_{r}^{e} .$ This assesses the improved precision of the model-based estimate when compared with the direct estimate of a geographic region, and due to the fact that the CV of the direct estimate is always larger than that of the model-based estimate, this quantity is always positive. We also examine the relative bias calculated as $\frac{{{\hat{p}}^{e}_{r} \text{ - }\;\hat{p}_{r}^{o} }}{{{\hat{p}}^{e}_{r} }}\; \times \;100{\% }$, where $\hat{p}_{r}^{e}$ is the model-based estimate of the prevalence of child developmental vulnerability and $\hat{p}_{r}^{o}$ the direct estimate. A positive bias reflects that the direct estimate is under-estimating the prevalence of child developmental vulnerability, while a negative bias shows that the direct estimate is over-estimating the true prevalence.

In Table 3, we present a set of summary statistics for the direct and model-based estimators for the prevalence of child developmental vulnerability in all five domains.

**Table 3:** Profile of direct and model-based estimated prevalence of child developmental vulnerability in regions in Australia

	Direct based	Model-based
For all five domains		
Number of SA3s with no valid samples	4	0
Range of prevalence estimates (in %)	[0.33, 47.06]	[1.52, 47.11]
Range of standard deviation of prevalence estimate (in %)	[0.31, 6.05]	[0.24, 4.00]
Range of CV (in %)	[4.36, 99.83]	[4.37, 27.12]
Range of 95% confidence (predictive) interval length (in %)	[1.23, 23.73]	[0.93, 15.61]
A. Physical Health and Wellbeing		
Number of SA3s with no valid samples	4	0
Range of prevalence estimates (in %)	[2.09, 35.29]	[2.91, 32.22]
Range of standard deviation of prevalence estimates (in %)	[0.43, 5.80]	[0.41, 3.53]
Range of CV (in %)	[4.78, 41.98]	[4.66, 20.57]
Range of 95% confidence (predictive) interval length (in %)	[1.68, 22.72]	[1.61, 13.80]
B. Social Competence		
Number of SA3s with no valid samples	4	0
Range of prevalence estimates (in %)	[2.85, 32.00]	[3.63,33.25]
Range of standard deviation of prevalence estimates (in %)	[0.46, 5.35]	[0.44, 3.32]
Range of CV (in %)	[4.36, 41.98]	[4.37, 19.29]
Range of 95% confidence (predictive) interval length (in %)	[1.81, 20.97]	[1.74, 12.92]
C. Emotional maturity		
Number of SA3s with no valid samples	4	0
Range of prevalence estimates (in %)	[3.25, 26.02]	[4.04, 26.75]
Range of standard deviation of prevalence estimates (in %)	[0.41, 5.75]	[0.40, 2.52]
Range of CV (in %)	[5.01, 40.05]	[4.85, 18.38]
Range of 95% confidence (predictive) interval length (in %)	[1.62, 22.54]	[1.55, 9.84]
D. Language and cognitive skills		
Number of SA3s with no valid samples	4	0
Range of prevalence estimates (in %)	[0.33, 47.06]	[1.52, 47.11]
Range of standard deviation of prevalence estimates (in %)	[0.31, 6.05]	[0.24, 4.00]
Range of CV (in %)	[5.00, 99.83]	[4.87, 27.12]
Range of 95% confidence (predictive) interval length (in %)	[1.23, 23.73]	[0.93, 15.61]
E. Communication skills		
Number of SA3s with no valid samples	4	0
Range of prevalence estimates (in %)	[1.14, 38.56]	[1.93, 39.51]
Range of standard deviation of prevalence estimates (in %)	[0.41, 5.44]	[1.93, 39.51]
Range of CV (in %)	[4.53, 69.01]	[0.05, 19.73]
Range of 95% confidence (predictive) interval length (in %)	[1.62, 21.33]	[0.40, 3.93]

For brevity purposes, we focus this discussion on two domains: physical health and wellbeing, and language and cognitive skills. (The rest of the results are in the Additional files 1, 2, 3, 4, 5 and 6). We present our results in the form of maps for better visualisation, since they have the benefits of providing a clear and concise summary of the data, easily identifying patterns, and allowing us to discern relationships. For the CV and bias maps presented, for comparability across all the different domains we use cutpoints based on the 20, 40, 60, 80 percentiles of the distribution of the CV difference and the relative bias, respectively. This allows us to visually examine the geographical variation in the estimates of the prevalence in child developmental vulnerability across regions of Australia, using a similar scale. What we notice in all domains, is that applying spatial modelling techniques lead to an improvement in the estimates of the regional prevalence of child vulnerability. Through examining the maps of the CV and relative bias, we can identify particular regions, such as Gascoyne and Esperance (both in Western Australia), Dural -Wisemans Ferry (in New South Wales), Caboolture Hinterlands (in Queensland) and South East Coast (in Tasmania), which are all sparsely populated regions, where there is marked difference between the model-based estimates and direct estimates. Note that, for the four areas with missing data, although the model-based procedure provides derived estimates of prevalence, the difference in CV and relative bias cannot be computed as the direct estimation has no value. As such, in our comparison between the model-based and direct estimates across Australia, we do not include these four areas.

**Fig. 1:**
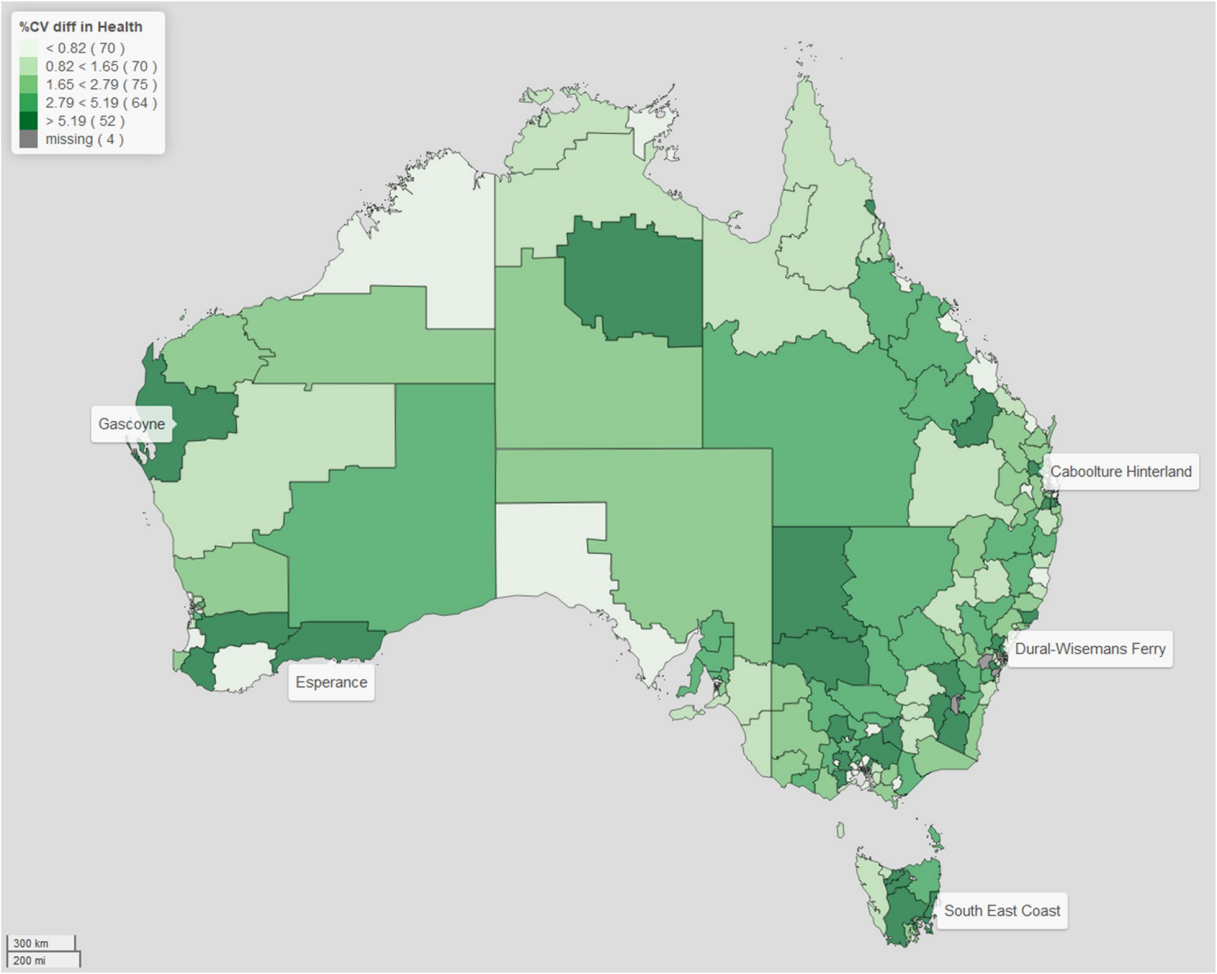
Map of the difference in CV in *Physical Health and Wellbeing* domain. The filling colours reflect the distribution of the difference between the percentage coefficient of variation (CV) of the model-based approach compared with direct estimation of the prevalence of vulnerability in the *Physical Health and Wellbeing* domain

**Fig. 2:**
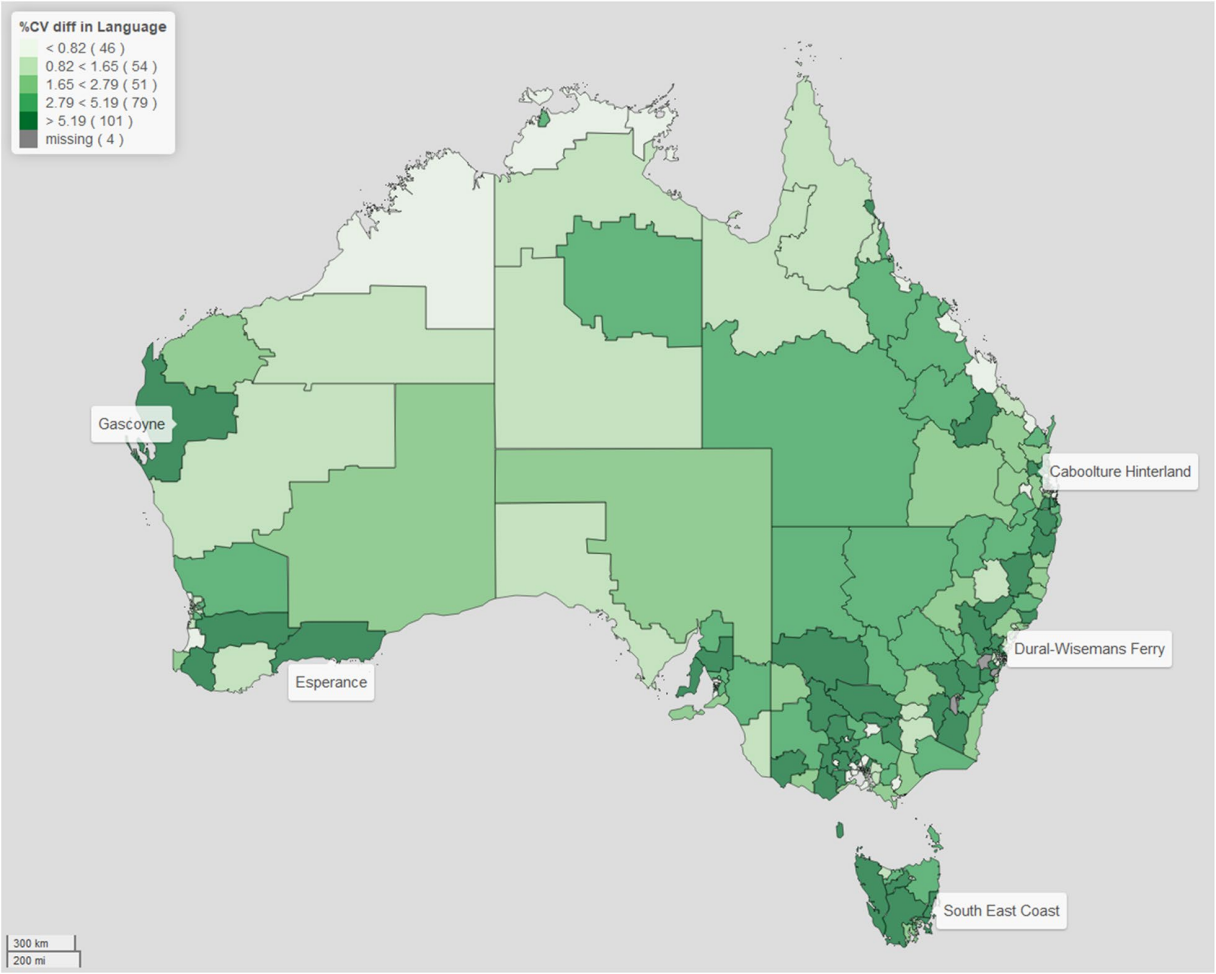
Map of the difference in CV in *Language and Cognitive Skills* domain. The filling colours reflect the distribution of the difference between the percentage coefficient of variation (CV) of the model-based approach compared with direct estimation of the prevalence of vulnerability in the *Language and Cognitive Skills* domain

For Figs. 1 and 2, the areas of dark green are regions with the greatest improvement in the CV when the model-based approach is compared with the direct estimation of the prevalence of child vulnerability for the different domains. This shows that on average the improvement is 2.9% for Health and Wellbeing domain and 4.3% for the Language and Cognitive Skills domain. In particular, it can be noticed that there is remarkable improvement in the estimates of the prevalence of children classified to be developmentally vulnerable in the Language and Cognitive Skills domain where almost a third of the regions are shown to have the largest improvement in the precision after model based estimation.

**Fig. 3:**
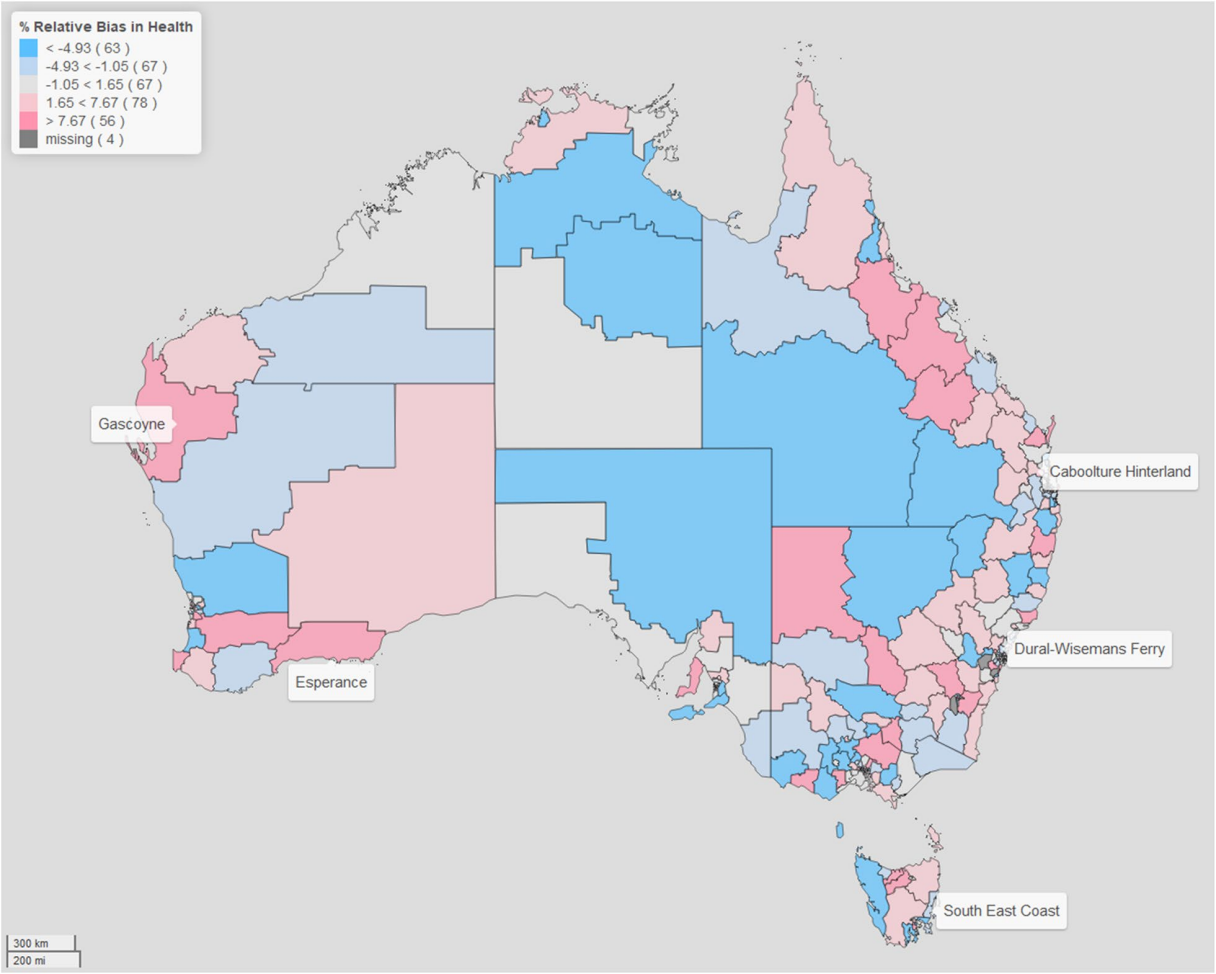
Map of the ratio of the relative bias in *Physical Health and Wellbeing* domain.The filling colours reflect the distribution of the ratio of the percentage relative bias (RB) of the model-based approach compared with direct estimation of the prevalence of vulnerability in the *Physical Health and Wellbeing* domain

**Fig. 4:**
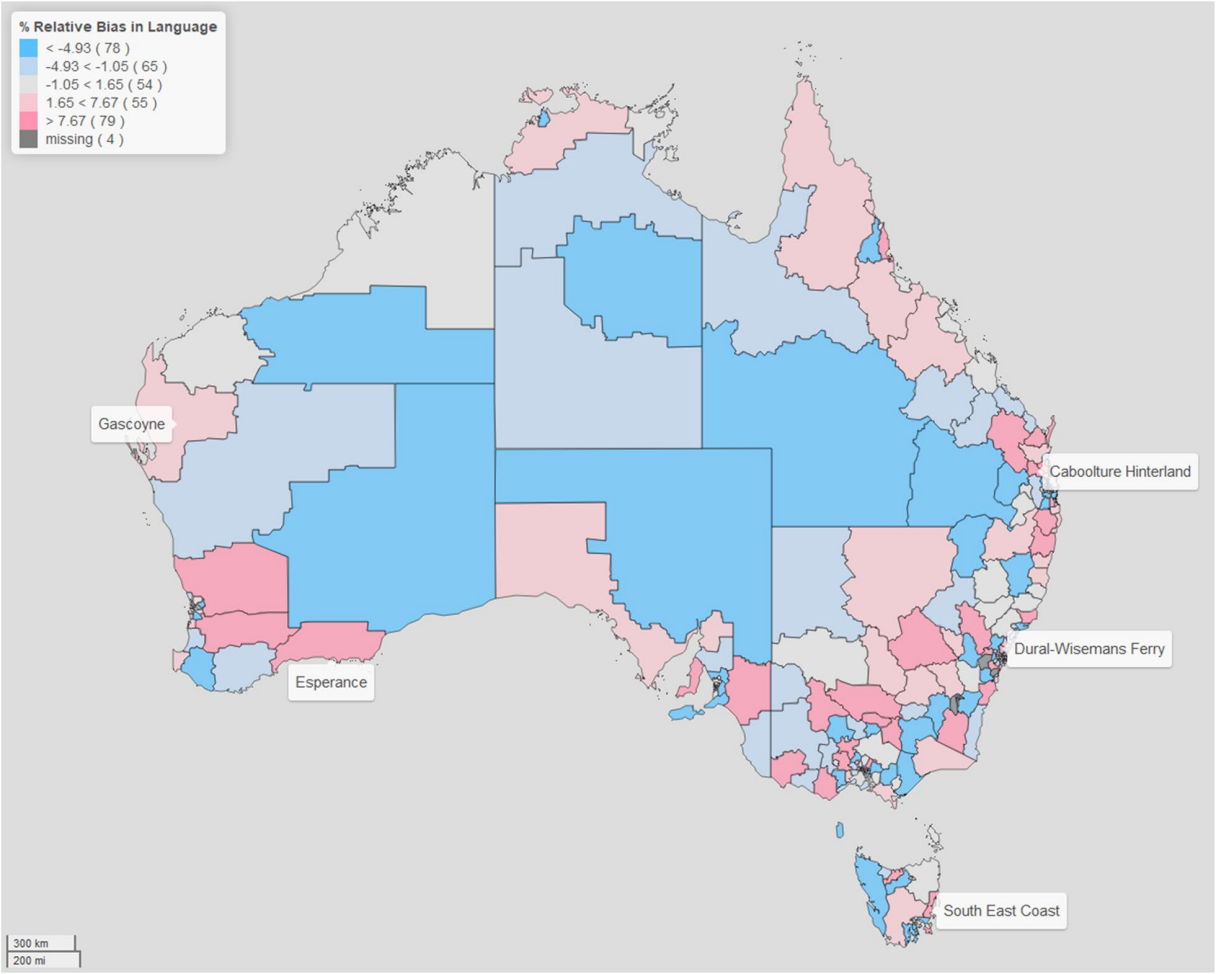
Map of the ratio of the relative bias in *Language and Cognitive Skills* domain. The filling colours reflect the distribution of the ratio of the percentage relative bias (RB) of the model-based approach compared with direct estimation of the prevalence of vulnerability in the *Language and Cognitive Skills* domain

In Figs. 3 and 4, we plot the percentage relative bias of the model-based estimation compared with the direct estimation. Note that in theory, the direct estimates are unbiased, and as such the model-based estimates derived from the fitted model should be consistent with the (unbiased) direct estimates. In essence, the model-based estimates should provide an approximation to the direct estimates that is consistent with these values being ‘close’ to the expected values of the direct estimates. We examine the distribution of over-estimation (where the model-based procedure produces larger estimates than using the direct approach), and under-estimation (where the opposite occurs). On average, the relative bias is 0.74% for Physical Health and Wellbeing domain, while it is 1.98% for the Language and Cognitive Skills domain. However, the maps provide a good way of examining the distribution of the percentage relative bias, and allows us to identify particular regions that differ significantly in their prevalence estimates under direct estimation and model-based approach. We also note that in the five identified sparsely populated regions there is evidence that the model-based estimation improves the reliability and generally provide a better indication of the level and spatial variation of child developmental vulnerability across Australia.

## Discussion and conclusion

Our paper contributes to the literature on early childhood development through identifying the impact of socio-economic disadvantage on geographic variation in developmental vulnerability. This is a strength of our analysis in that it brings together a measure of disadvantage with a smoothing process to provide novel insights into the spatial variation in early childhood developmental vulnerability. It also showcases the utility of statistical model-based methods in understanding the geographical patterns of child developmental vulnerability. These results show that the model-based estimates are more reliable, stable and useful for informing policy and planning to improve health and education outcomes at local regional level.

It is prudent to acknowledge some limitations in this analysis as well. Our analysis has a single covariate, relative disadvantage, used to explain variability in developmental vulnerability. There are many other factors, such as remoteness, community factors, selection effects and contextual influences that contribute to the observed geographical differences in children’s developmental vulnerability. Nonetheless, perhaps due to the way that relative disadvantage captures both the ecological factors and neighbourhood characteristics [6, 27] the small area statistical models are able to quantify the extent and characteristics of the geographic variation in the prevalence of vulnerability. Further, for reasons of simplicity, interpretability and model efficiency adding more variables introduces instability into small area estimation [14, 40].

Secondly, the AEDC has been conducted four times and our focus has been on the most recent collection, 2018. The data from 4 years could be pooled over time and a spatio-temporal data analysis carried out [65] allowing us to compare changes in child developmental vulnerability over both geography and time, but the changes in AEDC geographical boundaries over the different collections introduces some complexity in the data analysis. However, future work could implement this analysis, including data from a new collection planned for 2021 for maximum timeliness.

In conclusion, this paper examines the prevalence of early childhood developmental vulnerability, and specifically uses Bayesian spatial modelling to provide improved localised estimates of developmental vulnerability. Our results show that there is an important geographical dimension to developmental vulnerability in Australia, and that it is concentrated in particular areas. Through the use of this model, we borrow strength from neighbouring regions to improve the reliability and precision of child vulnerability. In addition, we are able to identify the associations between key area level factors and geographical patterns through incorporating the areal level of disadvantage. Areas experiencing high levels of relative socio-economic disadvantage, were more likely to exhibit higher levels of childhood developmental vulnerability, across all the domains. Following similar studies (e.g. [14] and [66]) we use a single summary measure of the socio-economic condition of a specific local area, since it is simple and interpretable, as well as model efficient because adding more variables introduces instability in the small area estimates [67]. Our findings using socio-economic disadvantage to characterize the spatial heterogeneity of geographical areas mirror those found in the United Kingdom [68], the United States [69], Norway [70], amongst other countries. Although the AEDC collects information from roughly 309,000 children representing 96% of the population of children in their first year of full-time education, there are a number of sparsely populated areas where the data is not reasonably large enough to provide reliable estimates of the relatively small prevalence of child vulnerability. The methods developed in this paper are therefore applicable to other countries with high variability in population density and sample effort. In addition, in low- and middle-income countries, the Multiple Indicator Cluster Surveys (MICS) run under the auspices of UNICEF collect information on child mortality, morbidity and health indicators which is used to measure the specific targets in the Millennium Development Goals and the Sustainable Development Goals. The surveys collect information from a sample of individuals and these are then extrapolated to the population but this can be problematic since the sample sizes are not large enough to provide detailed dis-aggregated small area information, and our small area spatial models can be applied in this context.

## Supplementary information

**Additional file 1:** Map of the difference in CV in *Social Competence* domain. The filling colours reflect the distribution of the difference between the percentage coefficient of variation (CV) of the model-based approach compared with direct estimation of the prevalence of vulnerability in the *Social Competence* domain.

**Additional file 2:** Map of the difference in CV in ***Emotional Maturity*** domain. The filling colours reflect the distribution of the difference between the percentage coefficient of variation (CV) of the model-based approach compared with direct estimation of the prevalence of vulnerability in the *Emotional Maturity* domain.

**Additional file 3:** Map of the difference in CV in *Communication Skills* domain. The filling colours reflect the distribution of the difference between the percentage coefficient of variation (CV) of the model-based approach compared with direct estimation of the prevalence of vulnerability in the *Communication Skills* domain.

**Additional file 4:** Map of the ratio of the relative bias in ***Social Competence*** domain. The filling colours reflect the distribution of the ratio of the percentage relative bias (RB) of the model-based approach compared with direct estimation of the prevalence of vulnerability in the *Social Competence* domain.

**Additional file 5:** Map of the ratio of the relative bias in *Emotional Maturity* domain. The filling colours reflect the distribution of the ratio of the percentage relative bias (RB) of the model-based approach compared with direct estimation of the prevalence of vulnerability in the *Emotional Maturity* domain.

**Additional file 6:** Map of the ratio of the relative bias in *Communication Skills* domain. The filling colours reflect the distribution of the ratio of the percentage relative bias (RB) of the model-based approach compared with direct estimation of the prevalence of vulnerability in the *Communication Skills* domain.

**Additional file 7:** Table of Crude and Model-based Estimators (in percentage) of Developmental Vulnerability in each Statistical Area Level 3 (SA3) region. Additional file 7: Table S1 shows the detailed crude and model-based estimators in SA3s. We also attached the standard deviations of each estimation in the brackets, to make it convenient for comparison
