# Supplementary figures and images for: Bayesian spatial modelling of early childhood development in Australian regions
Source: Int J Health Geogr. 2020 Oct 19;19:43. doi: 10.1186/s12942-020-00237-x (PMC7574340; doi:10.1186/s12942-020-00237-x)

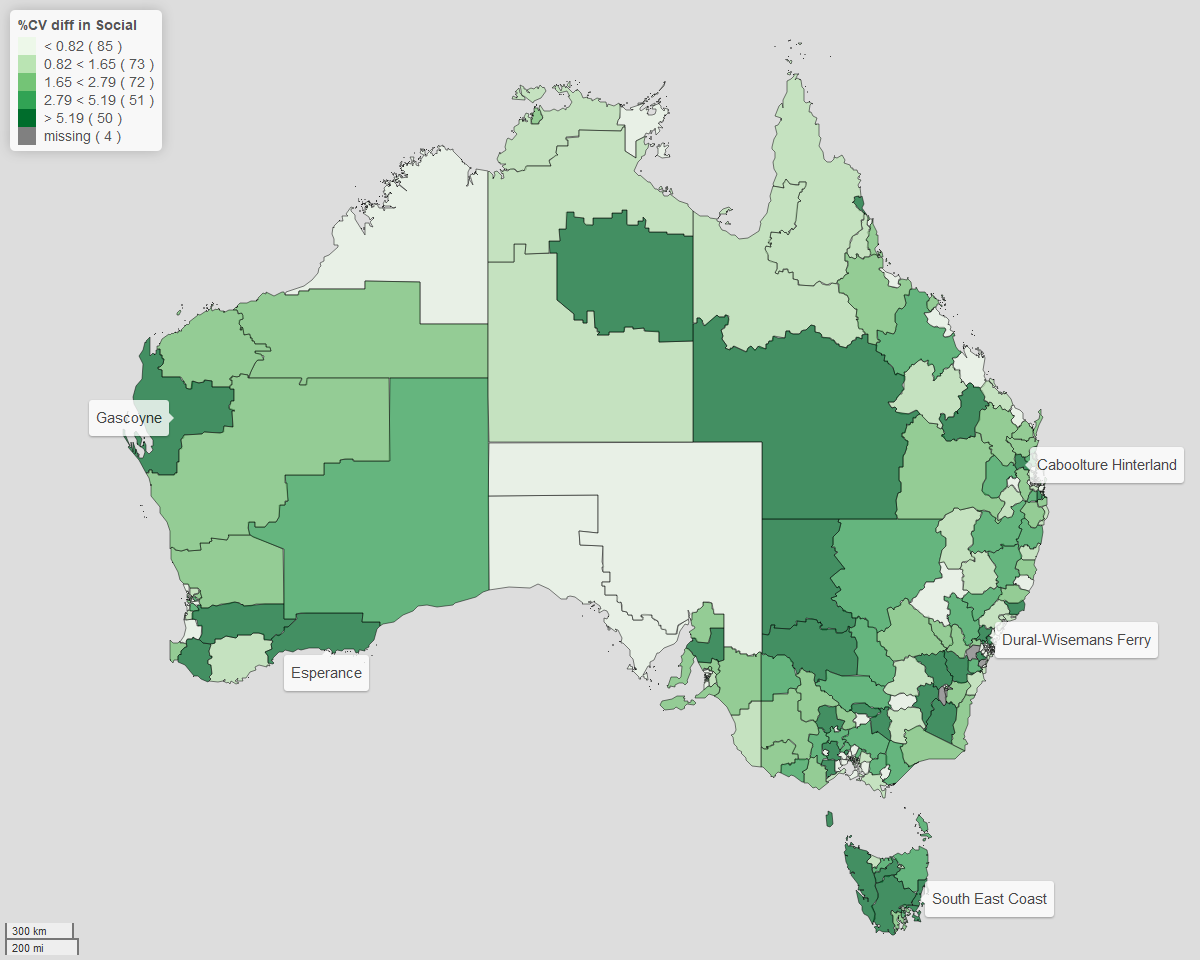

Supplement: Supplementary file 1 — Additional file 1: Map of the difference in CV in Social Competence domain. The filling colours reflect the distribution of the difference between the percentage coefficient of variation (CV) of the model-based approach compared with direct estimation of the prevalence of vulnerability in the Social Competence domain. [file 12942_2020_237_MOESM1_ESM.png]

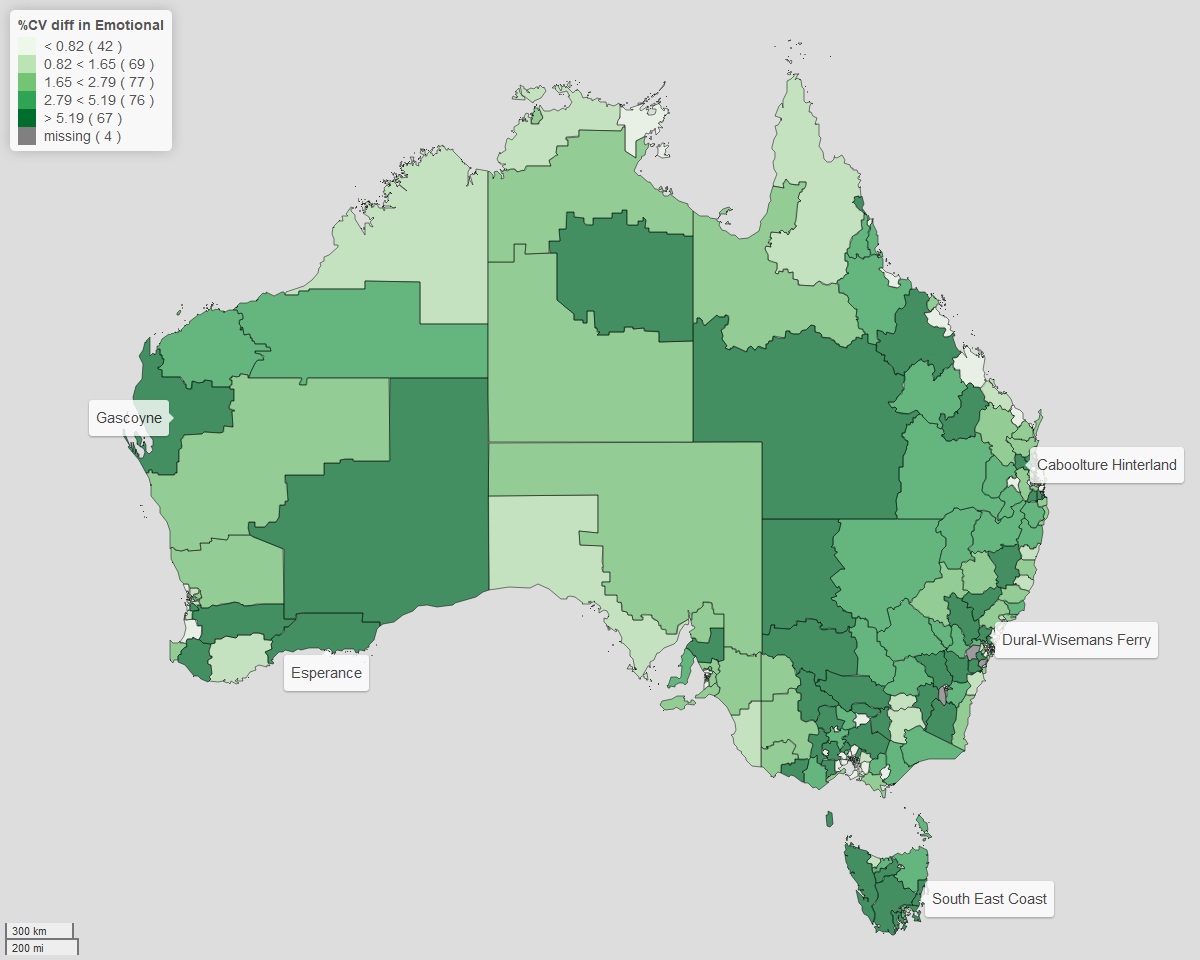

Supplement: Supplementary file 2 — Additional file 2: Map of the difference in CV in Emotional Maturity domain. The filling colours reflect the distribution of the difference between the percentage coefficient of variation (CV) of the model-based approach compared with direct estimation of the prevalence of vulnerability in the Emotional Maturity domain. [file 12942_2020_237_MOESM2_ESM.png]

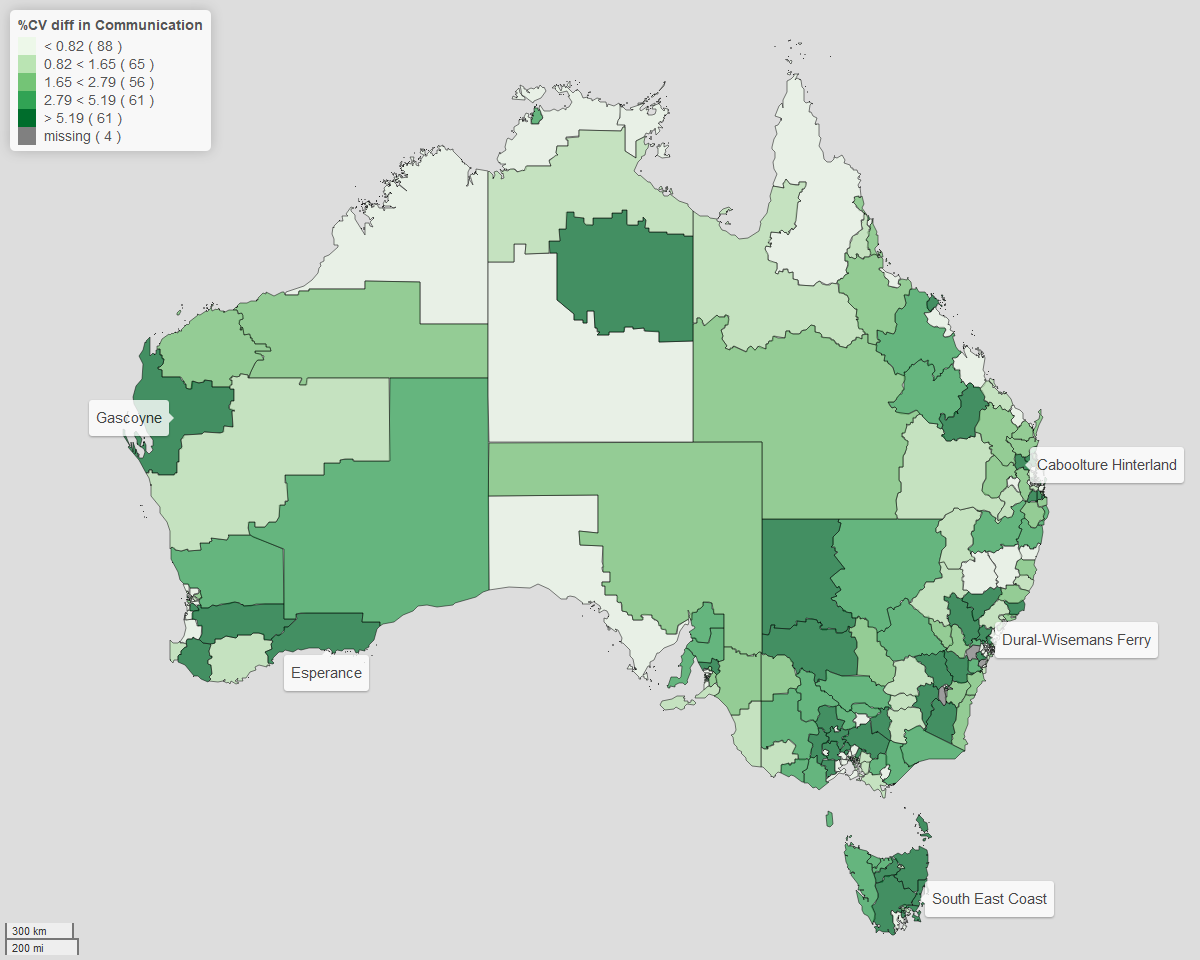

Supplement: Supplementary file 3 — Additional file 3: Map of the difference in CV in Communication Skills domain. The filling colours reflect the distribution of the difference between the percentage coefficient of variation (CV) of the model-based approach compared with direct estimation of the prevalence of vulnerability in the Communication Skills domain. [file 12942_2020_237_MOESM3_ESM.png]

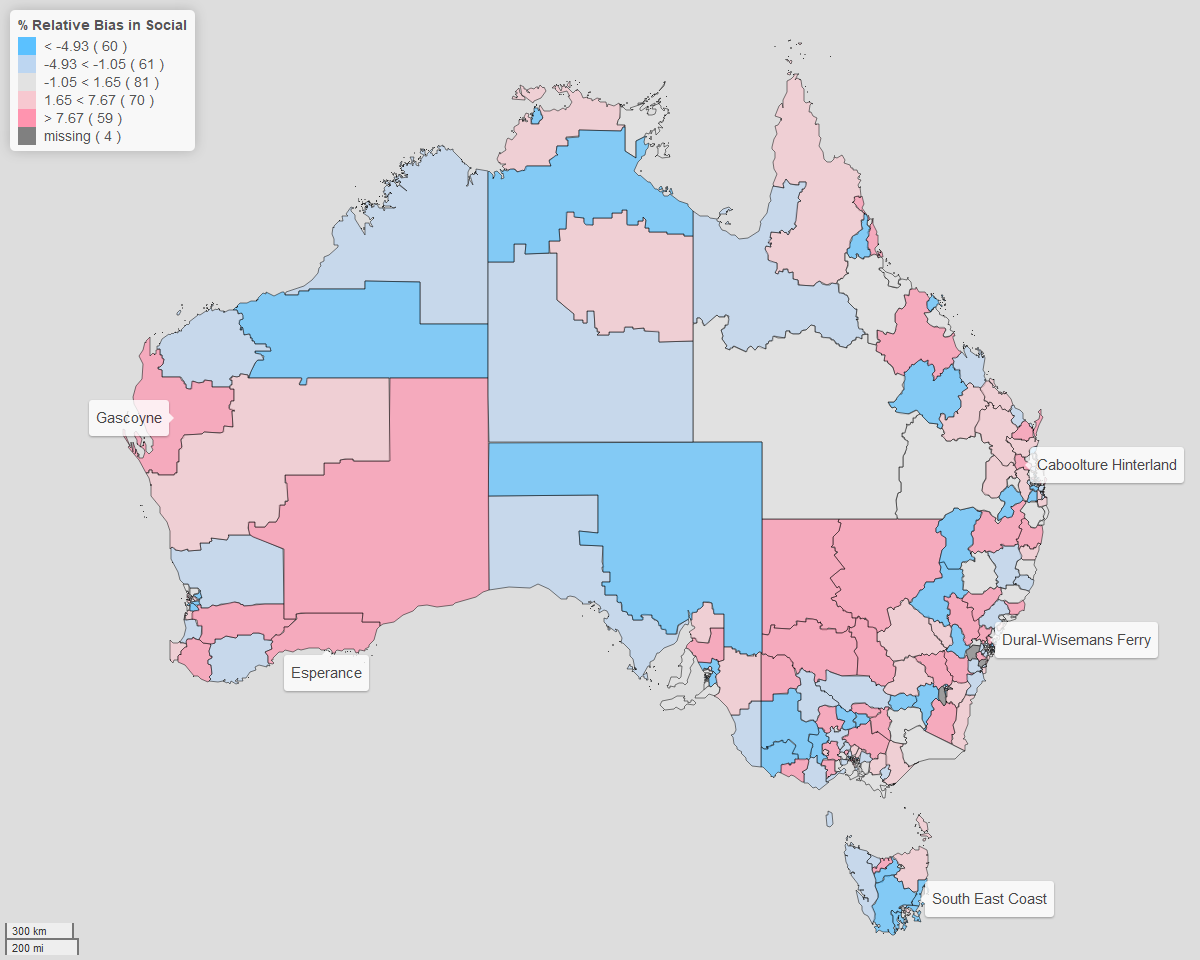

Supplement: Supplementary file 4 — Additional file 4: Map of the ratio of the relative bias in Social Competence domain. The filling colours reflect the distribution of the ratio of the percentage relative bias (RB) of the model-based approach compared with direct estimation of the prevalence of vulnerability in the Social Competence domain. [file 12942_2020_237_MOESM4_ESM.png]

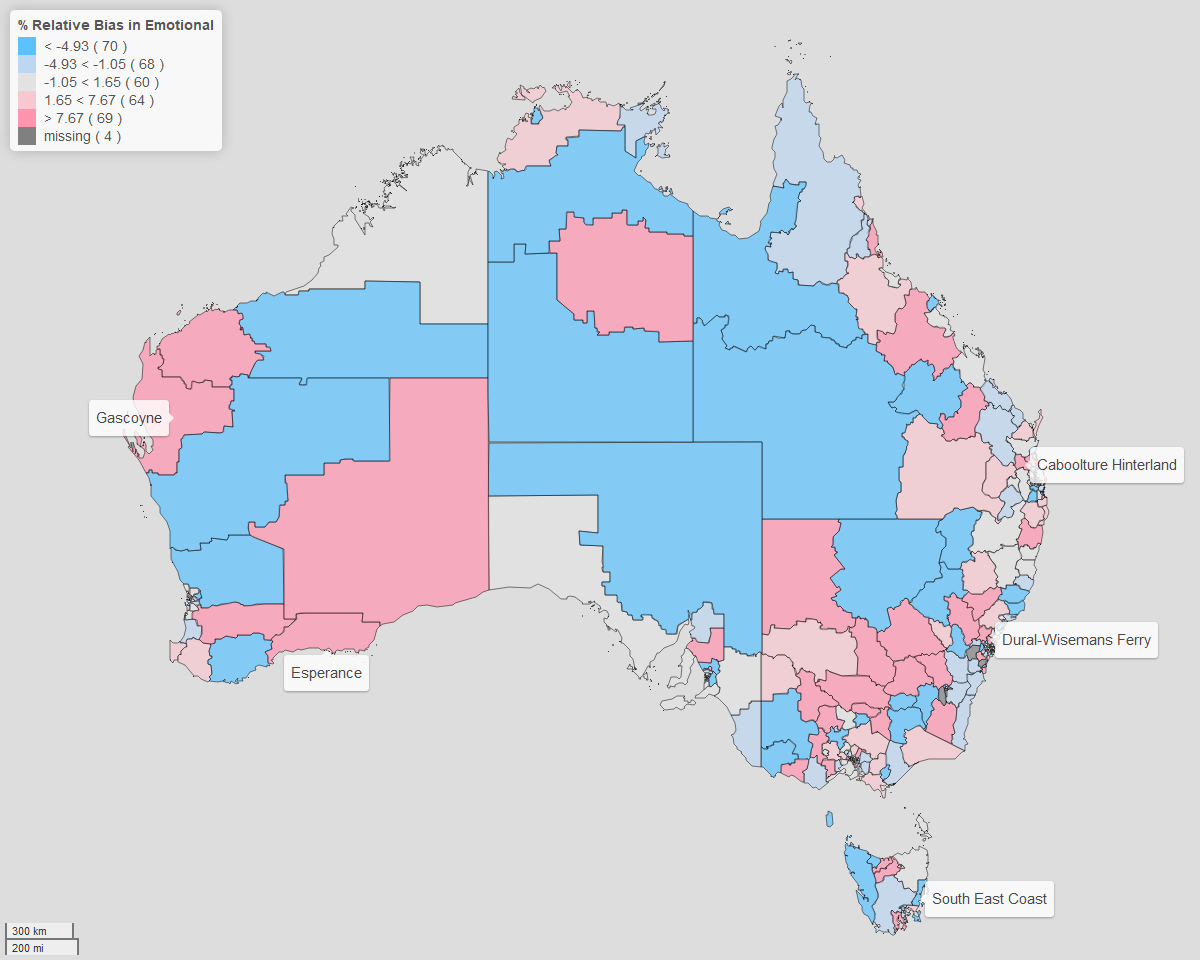

Supplement: Supplementary file 5 — Additional file 5: Map of the ratio of the relative bias in Emotional Maturity domain. The filling colours reflect the distribution of the ratio of the percentage relative bias (RB) of the model-based approach compared with direct estimation of the prevalence of vulnerability in the Emotional Maturity domain. [file 12942_2020_237_MOESM5_ESM.png]

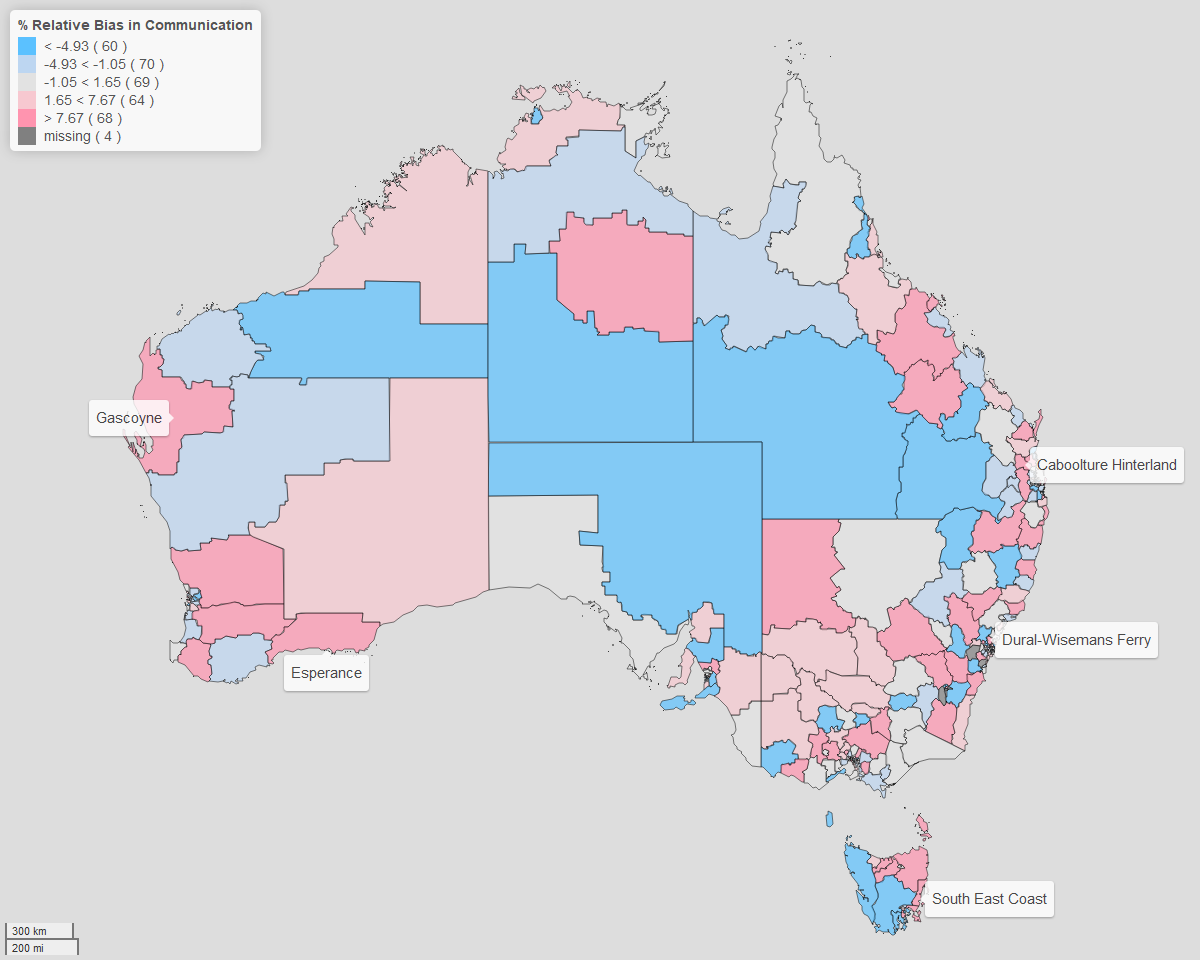

Supplement: Supplementary file 6 — Additional file 6: Map of the ratio of the relative bias in Communication Skills domain. The filling colours reflect the distribution of the ratio of the percentage relative bias (RB) of the model-based approach compared with direct estimation of the prevalence of vulnerability in the Communication Skills domain. [file 12942_2020_237_MOESM6_ESM.png]
