# Supplementary material for: Bayesian spatial modelling of early childhood development in Australian regions
Source: Int J Health Geogr. 2020 Oct 19;19:43. doi: 10.1186/s12942-020-00237-x (PMC7574340; doi:10.1186/s12942-020-00237-x)
Supplement: Supplementary file 7 — Additional file 7: Table of Crude and Model-based Estimators (in percentage) of Developmental Vulnerability in each Statistical Area Level 3 (SA3) region. Additional file 7: Table S1 shows the detailed crude and model-based estimators in SA3s. We also attached the standard deviations of each estimation in the brackets, to make it convenient for comparison [file 12942_2020_237_MOESM7_ESM.docx]

**Table S1. Crude and Model-based Estimators (in percentage) of Developmental Vulnerability in each Statistical Area Level 3 (SA3) region**

| **SA3 Code** | **Statistical Area Level 3 Name** | **Physical Health and Wellbeing** | | **Social Competence** | | **Emotional Maturity** | | **Language and Cognitive Skills** | | **Communication Skills** | |
| --- | --- | --- | --- | --- | --- | --- | --- | --- | --- | --- | --- |
|  |  | **Crude Estimation of Prevalence (standard deviation)** | **Model-based Estimation of Prevalence (standard deviation)** | **Crude Estimation of Prevalence (standard deviation)** | **Model-based Estimation of Prevalence (standard deviation)** | **Crude Estimation of Prevalence (standard deviation)** | **Model-based Estimation of Prevalence (standard deviation)** | **Crude Estimation of Prevalence (standard deviation)** | **Model-based Estimation of Prevalence (standard deviation)** | **Crude Estimation of Prevalence (standard deviation)** | **Model-based Estimation of Prevalence (standard deviation)** |
| ***New South Wales*** | | | | | | | | | | | |
| 10102 | Queanbeyan | 7.49(0.95) | 8.4(0.73) | 8.14(0.98) | 8.42(0.79) | 8.67(1.01) | 8.32(0.71) | 5.95(0.85) | 5.54(0.57) | 6.59(0.89) | 6.19(0.68) |
| 10103 | Snowy Mountains | 11(2.16) | 10.56(1.35) | 3.83(1.33) | 7.68(1.12) | 5.26(1.54) | 7.88(1.04) | 3.83(1.33) | 5.99(0.92) | 2.39(1.06) | 5.27(0.95) |
| 10104 | South Coast | 9.71(1.14) | 10.11(0.98) | 9.12(1.1) | 9.5(0.96) | 9.43(1.12) | 9.32(0.89) | 7.96(1.04) | 7.85(0.86) | 6.91(0.97) | 7.08(0.85) |
| 10105 | Goulburn - Mulwaree | 9.69(1.44) | 9.89(1.01) | 7.09(1.25) | 8.63(1) | 8.31(1.35) | 8.22(0.85) | 7.09(1.25) | 7.06(0.84) | 5.91(1.15) | 6.82(0.88) |
| 10106 | Young - Yass | 8.25(1.26) | 9.67(0.94) | 7.4(1.2) | 8.89(0.94) | 6.13(1.1) | 7.75(0.77) | 6.34(1.12) | 6.78(0.75) | 5.29(1.03) | 6.48(0.82) |
| 10201 | Gosford | 8.58(0.63) | 8.56(0.58) | 8.12(0.61) | 8.13(0.58) | 6.87(0.57) | 6.88(0.52) | 3.96(0.44) | 4.03(0.39) | 5.21(0.5) | 5.3(0.46) |
| 10202 | Wyong | 11.07(0.7) | 10.93(0.66) | 11.93(0.73) | 11.64(0.68) | 10.03(0.67) | 9.72(0.62) | 7.35(0.59) | 7.05(0.54) | 6.95(0.57) | 6.97(0.54) |
| 10301 | Bathurst | 10.77(1.31) | 10.08(0.98) | 11.31(1.34) | 10.28(1.04) | 8.44(1.18) | 7.7(0.8) | 6.46(1.04) | 5.88(0.72) | 8.8(1.2) | 7.87(0.94) |
| 10302 | Lachlan Valley | 9.42(1.09) | 10.18(0.87) | 9.56(1.09) | 10.02(0.9) | 6.52(0.92) | 7.59(0.7) | 6.23(0.9) | 7.17(0.72) | 6.37(0.91) | 7.29(0.78) |
| 10303 | Lithgow - Mudgee | 10.41(1.35) | 10.69(1) | 7.86(1.19) | 9.07(0.96) | 6.29(1.08) | 7.28(0.75) | 4.91(0.96) | 6.08(0.73) | 5.11(0.98) | 6.6(0.84) |
| 10304 | Orange | 8.77(0.99) | 8.91(0.85) | 7.31(0.91) | 7.86(0.79) | 6.09(0.83) | 6.54(0.68) | 4.99(0.76) | 5.11(0.62) | 6.21(0.84) | 6.3(0.73) |
| 10401 | Clarence Valley | 7.54(1.18) | 9.32(0.99) | 7.94(1.2) | 9.14(1.01) | 7.37(1.17) | 8.2(0.88) | 4.77(0.95) | 5.9(0.76) | 7.34(1.16) | 8.19(0.99) |
| 10402 | Coffs Harbour | 8.05(0.88) | 8.55(0.81) | 7.22(0.84) | 7.69(0.77) | 8.16(0.89) | 8.1(0.78) | 4.92(0.7) | 5.02(0.6) | 8.27(0.89) | 8.16(0.82) |
| 10501 | Bourke - Cobar - Coonamble | 16.51(2.05) | 15.71(1.44) | 11.01(1.73) | 12.44(1.34) | 13.5(1.89) | 11.79(1.16) | 11.96(1.8) | 12.46(1.29) | 12.54(1.83) | 12.59(1.4) |
| 10502 | Broken Hill and Far West | 12.3(2.07) | 13.79(1.53) | 10.71(1.95) | 12.14(1.45) | 9.13(1.81) | 10.8(1.25) | 14.29(2.2) | 14(1.61) | 5.53(1.44) | 8.74(1.28) |
| 10503 | Dubbo | 10.52(0.99) | 10.7(0.83) | 13.85(1.11) | 12.95(0.96) | 9.41(0.94) | 8.87(0.72) | 7.4(0.84) | 7.29(0.68) | 9.27(0.94) | 9.04(0.81) |
| 10601 | Lower Hunter | 10.44(0.89) | 10.45(0.71) | 10.44(0.89) | 10.28(0.76) | 7.93(0.79) | 8.16(0.61) | 6.49(0.71) | 6.46(0.54) | 7.49(0.76) | 7.47(0.64) |
| 10602 | Maitland | 7.91(0.8) | 8.12(0.73) | 8.79(0.84) | 8.78(0.76) | 7.3(0.77) | 7.4(0.68) | 5.58(0.68) | 5.46(0.6) | 6.15(0.71) | 6.19(0.65) |
| 10603 | Port Stephens | 8.94(1.03) | 8.93(0.87) | 9.07(1.03) | 8.99(0.88) | 8.87(1.03) | 8.52(0.82) | 6.1(0.86) | 5.74(0.68) | 6.61(0.89) | 6.48(0.76) |
| 10604 | Upper Hunter | 10.8(1.57) | 10.83(1.12) | 8.74(1.43) | 9.57(1.08) | 5.47(1.16) | 7.23(0.82) | 6.7(1.27) | 6.72(0.84) | 6.94(1.29) | 7.61(0.99) |
| 10701 | Dapto - Port Kembla | 14.49(1.12) | 13.5(1.01) | 12.68(1.06) | 12.1(0.96) | 8.77(0.9) | 8.3(0.78) | 7.75(0.85) | 7.39(0.75) | 11.27(1) | 10.85(0.92) |
| 10702 | Illawarra Catchment Reserve | NA | 8.11(1.35) | NA | 8.37(1.51) | NA | 6.49(1.02) | NA | 4.85(0.92) | NA | 6.75(1.45) |
| 10703 | Kiama - Shellharbour | 7.29(0.77) | 7.68(0.69) | 6.94(0.75) | 7.39(0.68) | 5.26(0.66) | 5.85(0.58) | 4.77(0.63) | 4.91(0.55) | 6.68(0.74) | 6.7(0.67) |
| 10704 | Wollongong | 5.61(0.59) | 6.25(0.54) | 6.61(0.64) | 7(0.58) | 5.94(0.61) | 6.06(0.52) | 3.01(0.44) | 3.5(0.39) | 5.34(0.58) | 5.61(0.54) |
| 10801 | Great Lakes | 7.89(1.65) | 9.48(1.34) | 7.14(1.58) | 8.83(1.27) | 10.53(1.88) | 9.86(1.29) | 3.76(1.17) | 5.7(0.99) | 3.01(1.05) | 5.42(1.02) |
| 10802 | Kempsey - Nambucca | 16.29(1.6) | 15.37(1.39) | 11.99(1.41) | 12.02(1.21) | 9.55(1.27) | 9.59(1.03) | 7.3(1.13) | 7.75(0.98) | 7.68(1.15) | 8.66(1.06) |
| 10804 | Port Macquarie | 9.26(0.97) | 9.6(0.88) | 11.72(1.07) | 11.21(0.95) | 8.48(0.93) | 8.38(0.8) | 5.36(0.75) | 5.34(0.64) | 7.6(0.89) | 7.51(0.81) |
| 10805 | Taree - Gloucester | 13.3(1.43) | 12.71(1.13) | 10.82(1.31) | 10.91(1.07) | 10.82(1.31) | 10(0.96) | 7.65(1.12) | 7.61(0.87) | 7.8(1.13) | 8.2(0.97) |
| 10901 | Albury | 11.87(1.18) | 11.54(1.02) | 14(1.27) | 13.05(1.09) | 10.96(1.14) | 10.12(0.92) | 8.54(1.02) | 8.26(0.86) | 9.33(1.06) | 8.88(0.92) |
| 10902 | Lower Murray | 12.27(2.57) | 11.73(1.43) | 6.13(1.88) | 9.45(1.31) | 9.2(2.26) | 9.63(1.17) | 9.2(2.26) | 9.15(1.24) | 7.36(2.05) | 7.8(1.24) |
| 10903 | Upper Murray exc. Albury | 11.94(1.46) | 11.07(1) | 10.73(1.39) | 10.45(1.02) | 5.69(1.04) | 7.66(0.75) | 6.48(1.11) | 7.13(0.76) | 6.68(1.12) | 7.06(0.83) |
| 11001 | Armidale | 12.82(1.61) | 10.91(1.05) | 9.56(1.42) | 9.27(1.01) | 7.69(1.29) | 7.71(0.79) | 6.99(1.23) | 5.67(0.68) | 13.29(1.64) | 10.23(1.2) |
| 11002 | Inverell - Tenterfield | 13.23(1.6) | 13.72(1.19) | 10.76(1.47) | 11.76(1.16) | 9.87(1.41) | 9.99(0.94) | 8.52(1.32) | 9.12(0.94) | 9.42(1.38) | 10.51(1.14) |
| 11003 | Moree - Narrabri | 16.89(1.94) | 15.05(1.45) | 18.77(2.02) | 15.96(1.56) | 11.62(1.67) | 10.43(1.11) | 10.75(1.61) | 9.84(1.14) | 14.25(1.81) | 12.85(1.44) |
| 11004 | Tamworth - Gunnedah | 9.29(0.87) | 9.87(0.78) | 10.63(0.92) | 10.72(0.82) | 7.06(0.77) | 7.56(0.65) | 7.16(0.77) | 7.17(0.66) | 9.56(0.88) | 9.51(0.81) |
| 11101 | Lake Macquarie - East | 7.38(0.7) | 7.7(0.66) | 7.81(0.72) | 7.97(0.67) | 6.67(0.67) | 6.98(0.61) | 3.3(0.48) | 3.67(0.43) | 6.02(0.64) | 6.02(0.58) |
| 11102 | Lake Macquarie - West | 10.59(1.04) | 10.25(0.9) | 8.54(0.94) | 8.71(0.82) | 10.16(1.02) | 9.4(0.83) | 5.47(0.77) | 5.34(0.62) | 6.15(0.81) | 6.26(0.71) |
| 11103 | Newcastle | 9.41(0.67) | 9.26(0.61) | 8.74(0.64) | 8.7(0.59) | 6.97(0.58) | 7.16(0.52) | 4.64(0.48) | 4.68(0.43) | 7.28(0.59) | 7.08(0.55) |
| 11201 | Richmond Valley - Coastal | 6.78(0.86) | 7.25(0.77) | 8.18(0.94) | 8.27(0.83) | 6.71(0.86) | 6.87(0.72) | 3.56(0.64) | 3.72(0.53) | 4.32(0.7) | 4.8(0.64) |
| 11202 | Richmond Valley - Hinterland | 15.49(1.32) | 14.2(1.07) | 11.21(1.15) | 11.4(0.96) | 8.71(1.03) | 9.05(0.8) | 4.67(0.77) | 6.03(0.65) | 9.48(1.07) | 9.5(0.91) |
| 11203 | Tweed Valley | 7.85(0.86) | 8.4(0.75) | 8.15(0.87) | 8.67(0.78) | 6.65(0.8) | 7.17(0.66) | 4.49(0.66) | 4.82(0.55) | 6.22(0.77) | 6.55(0.68) |
| 11301 | Griffith - Murrumbidgee (West) | 7.75(1.01) | 9.08(0.86) | 8.03(1.03) | 8.85(0.88) | 7.03(0.97) | 7.81(0.76) | 7.17(0.98) | 7.58(0.78) | 7.46(1) | 7.6(0.82) |
| 11302 | Tumut - Tumbarumba | 11.64(2.33) | 12.12(1.38) | 14.29(2.55) | 12.52(1.52) | 12.23(2.39) | 10.2(1.17) | 10.58(2.24) | 9.61(1.22) | 8.99(2.08) | 8.84(1.3) |
| 11303 | Wagga Wagga | 9.07(0.85) | 9.34(0.74) | 7.93(0.8) | 8.45(0.72) | 5.47(0.68) | 6.5(0.58) | 5.64(0.68) | 6.05(0.59) | 7.31(0.77) | 7.26(0.67) |
| 11401 | Shoalhaven | 9.06(0.9) | 9.29(0.79) | 10.34(0.96) | 10.21(0.84) | 8.78(0.89) | 8.59(0.73) | 5.33(0.71) | 6(0.63) | 5.71(0.73) | 6.26(0.66) |
| 11402 | Southern Highlands | 7.3(1.16) | 7.24(0.82) | 7.69(1.18) | 7.54(0.87) | 6.35(1.09) | 6.28(0.71) | 5.33(1) | 4.54(0.61) | 5.92(1.05) | 5.57(0.77) |
| 11501 | Baulkham Hills | 5.92(0.52) | 5.97(0.44) | 5.72(0.51) | 5.9(0.46) | 5.05(0.49) | 5.11(0.4) | 2.05(0.31) | 2.21(0.24) | 5.43(0.5) | 5.37(0.44) |
| 11502 | Dural - Wisemans Ferry | 6.69(1.44) | 6.5(0.87) | 5.35(1.3) | 5.87(0.84) | 4.68(1.22) | 5.21(0.69) | 0.33(0.33) | 2.15(0.38) | 3.01(0.99) | 4.02(0.68) |
| 11503 | Hawkesbury | 6.49(1.4) | 6.85(0.82) | 5.84(1.34) | 6.43(0.85) | 3.25(1.01) | 5.18(0.63) | 3.57(1.06) | 3.18(0.47) | 6.49(1.4) | 5.17(0.8) |
| 11504 | Rouse Hill - McGraths Hill | 6.43(1) | 6.25(0.73) | 5.93(0.96) | 6.03(0.72) | 6.29(0.99) | 5.57(0.64) | 3.79(0.77) | 2.96(0.44) | 5.11(0.89) | 4.86(0.68) |
| 11601 | Blacktown | 9.92(0.68) | 9.81(0.62) | 10.64(0.7) | 10.59(0.65) | 7.3(0.59) | 7.18(0.52) | 7.3(0.59) | 6.99(0.53) | 10.28(0.69) | 10.28(0.65) |
| 11602 | Blacktown - North | 7.61(0.63) | 7.32(0.54) | 8.87(0.68) | 8.48(0.61) | 5.75(0.56) | 5.7(0.46) | 3.23(0.42) | 3.29(0.34) | 7.74(0.64) | 7.28(0.57) |
| 11603 | Mount Druitt | 11.57(0.73) | 11.66(0.69) | 13.14(0.77) | 13.14(0.72) | 9.11(0.66) | 8.86(0.58) | 10.9(0.71) | 10.72(0.66) | 11.63(0.73) | 11.86(0.7) |
| 11701 | Botany | 8.43(1.34) | 8.38(1) | 11.24(1.53) | 10.1(1.17) | 8.2(1.33) | 6.95(0.86) | 4.67(1.02) | 4.49(0.69) | 8.43(1.34) | 8.04(1.08) |
| 11702 | Marrickville - Sydenham - Petersham | 9.84(1.35) | 8.13(0.89) | 5.33(1.02) | 6.54(0.8) | 5.74(1.05) | 5.53(0.65) | 2.05(0.64) | 2.97(0.44) | 3.89(0.88) | 5.32(0.74) |
| 11703 | Sydney Inner City | 7.08(0.82) | 7.08(0.68) | 8.09(0.87) | 8.07(0.75) | 6.47(0.78) | 6.3(0.62) | 4.35(0.65) | 3.79(0.47) | 6.38(0.78) | 6.26(0.67) |
| 11801 | Eastern Suburbs - North | 6.65(0.73) | 6.36(0.66) | 9.33(0.85) | 8.76(0.77) | 7.35(0.77) | 6.79(0.67) | 1.21(0.32) | 1.6(0.29) | 3.54(0.54) | 3.64(0.5) |
| 11802 | Eastern Suburbs - South | 5.38(0.62) | 5.71(0.57) | 5.97(0.65) | 6.41(0.61) | 4.34(0.56) | 4.89(0.51) | 3.36(0.49) | 3.2(0.41) | 5(0.6) | 5.09(0.55) |
| 11901 | Bankstown | 8.47(0.57) | 8.54(0.51) | 10.34(0.62) | 10.41(0.58) | 5.37(0.46) | 5.71(0.4) | 6.31(0.5) | 6.36(0.44) | 12.13(0.67) | 12.1(0.64) |
| 11902 | Canterbury | 9.01(0.67) | 9.21(0.62) | 12.28(0.77) | 12.08(0.72) | 5.54(0.54) | 5.85(0.48) | 7.36(0.61) | 7.23(0.56) | 12.94(0.79) | 12.9(0.75) |
| 11903 | Hurstville | 7.86(0.71) | 7.89(0.64) | 8.91(0.75) | 8.83(0.68) | 5.7(0.62) | 5.58(0.52) | 4.81(0.56) | 4.64(0.48) | 10.65(0.81) | 10.32(0.76) |
| 11904 | Kogarah - Rockdale | 10.3(0.79) | 9.87(0.72) | 8.94(0.74) | 8.9(0.68) | 5.37(0.59) | 5.49(0.5) | 5.01(0.57) | 4.81(0.49) | 9.63(0.77) | 9.43(0.71) |
| 12001 | Canada Bay | 4.63(0.68) | 4.68(0.6) | 6.21(0.78) | 6.26(0.7) | 5.68(0.75) | 5.41(0.64) | 2.53(0.51) | 2.47(0.41) | 6.21(0.78) | 6.26(0.73) |
| 12002 | Leichhardt | 3.43(0.69) | 4.26(0.6) | 4.43(0.78) | 5.05(0.68) | 4.01(0.74) | 4.44(0.59) | 1(0.38) | 1.71(0.32) | 3.57(0.7) | 4.02(0.61) |
| 12003 | Strathfield - Burwood - Ashfield | 5.65(0.58) | 5.84(0.5) | 7.87(0.68) | 7.8(0.6) | 5.68(0.58) | 5.51(0.47) | 3.3(0.45) | 3.31(0.36) | 10.03(0.76) | 9.55(0.69) |
| 12101 | Chatswood - Lane Cove | 5.9(0.6) | 5.91(0.55) | 5.07(0.56) | 5.39(0.52) | 6.42(0.62) | 6.2(0.55) | 2.5(0.4) | 2.46(0.33) | 6.28(0.61) | 6.18(0.58) |
| 12102 | Hornsby | 7.37(0.76) | 7.18(0.66) | 6.02(0.69) | 6.27(0.61) | 5.83(0.69) | 5.82(0.57) | 2.88(0.49) | 2.7(0.37) | 5.09(0.64) | 5.2(0.58) |
| 12103 | Ku-ring-gai | 4.58(0.52) | 4.86(0.46) | 7.17(0.64) | 6.81(0.57) | 6.01(0.59) | 5.74(0.49) | 1.73(0.32) | 1.84(0.25) | 4.08(0.49) | 4.24(0.44) |
| 12104 | North Sydney - Mosman | 6.37(0.84) | 6.25(0.78) | 6.96(0.87) | 6.72(0.8) | 5.31(0.77) | 5.4(0.71) | 2.59(0.55) | 2.49(0.48) | 4.48(0.71) | 4.61(0.67) |
| 12201 | Manly | 2.09(0.62) | 2.91(0.6) | 2.85(0.73) | 3.63(0.7) | 3.61(0.81) | 4.04(0.72) | 1.14(0.46) | 1.52(0.41) | 1.14(0.46) | 1.93(0.5) |
| 12202 | Pittwater | 4.42(0.74) | 4.57(0.68) | 10.14(1.09) | 9.5(1.01) | 6.64(0.9) | 6.36(0.81) | 3.9(0.7) | 3.53(0.6) | 2.47(0.56) | 2.77(0.52) |
| 12203 | Warringah | 5.92(0.54) | 5.66(0.49) | 5.77(0.53) | 5.89(0.5) | 5.21(0.5) | 5.28(0.46) | 2.42(0.35) | 2.45(0.31) | 4.99(0.49) | 4.73(0.45) |
| 12301 | Camden | 4.85(0.69) | 5.32(0.61) | 4.85(0.69) | 5.55(0.63) | 5.78(0.75) | 5.64(0.62) | 2.16(0.47) | 2.68(0.4) | 2.89(0.54) | 3.57(0.52) |
| 12302 | Campbelltown (NSW) | 11.22(0.65) | 10.76(0.6) | 13(0.69) | 12.63(0.66) | 8.03(0.56) | 7.76(0.49) | 6.92(0.52) | 6.77(0.47) | 11.52(0.66) | 11.21(0.63) |
| 12303 | Wollondilly | 5.19(0.92) | 6.14(0.66) | 5.02(0.91) | 6.29(0.73) | 3.99(0.81) | 5.27(0.56) | 3.29(0.74) | 3.6(0.46) | 3.29(0.74) | 4.45(0.6) |
| 12401 | Blue Mountains | 8.7(0.99) | 8.1(0.76) | 7.48(0.92) | 7.41(0.75) | 6.5(0.86) | 6.2(0.61) | 2.57(0.55) | 3.24(0.43) | 4.66(0.74) | 4.81(0.6) |
| 12402 | Blue Mountains - South | NA | 8.48(1.3) | NA | 8.6(1.45) | NA | 6.89(0.99) | NA | 5.15(0.89) | NA | 6.41(1.3) |
| 12403 | Penrith | 7.9(0.6) | 7.95(0.52) | 9.8(0.66) | 9.59(0.59) | 7.56(0.59) | 7.11(0.48) | 5.85(0.52) | 5.51(0.43) | 6.83(0.56) | 6.85(0.51) |
| 12404 | Richmond - Windsor | 5.46(1.11) | 7.36(0.88) | 5.94(1.15) | 7.52(0.95) | 3.57(0.91) | 5.66(0.71) | 4.99(1.06) | 5.25(0.72) | 3.56(0.9) | 5.52(0.82) |
| 12405 | St Marys | 15.45(1.27) | 14.1(1.13) | 13.1(1.19) | 12.59(1.05) | 9.57(1.04) | 8.77(0.86) | 10.02(1.06) | 9.29(0.92) | 13.35(1.2) | 12.74(1.09) |
| 12501 | Auburn | 6.63(0.77) | 7.49(0.66) | 8.72(0.87) | 9.4(0.79) | 5.69(0.71) | 6.23(0.58) | 4.74(0.65) | 5.45(0.56) | 11.93(1) | 12.2(0.93) |
| 12502 | Carlingford | 6.66(0.8) | 6.85(0.66) | 7.8(0.87) | 7.93(0.73) | 5.58(0.74) | 5.71(0.58) | 3.33(0.58) | 3.36(0.43) | 9.26(0.94) | 9.04(0.83) |
| 12503 | Merrylands - Guildford | 12.76(0.7) | 12.37(0.65) | 13.42(0.71) | 13.3(0.68) | 7.07(0.54) | 7.13(0.48) | 9.82(0.62) | 9.57(0.57) | 16.22(0.77) | 16(0.75) |
| 12504 | Parramatta | 7.49(0.55) | 7.49(0.5) | 11.13(0.66) | 10.78(0.62) | 6.09(0.5) | 6.04(0.45) | 4.08(0.41) | 4.08(0.36) | 9.6(0.62) | 9.51(0.59) |
| 12601 | Pennant Hills - Epping | 8.13(1.04) | 7.12(0.76) | 8.85(1.08) | 7.87(0.84) | 6.97(0.97) | 6.1(0.65) | 2.03(0.54) | 2.19(0.34) | 6.24(0.92) | 6.04(0.74) |
| 12602 | Ryde - Hunters Hill | 6.37(0.59) | 6.43(0.53) | 6.49(0.59) | 6.66(0.55) | 4.69(0.51) | 5.1(0.46) | 2.72(0.39) | 2.74(0.33) | 8.63(0.68) | 8.4(0.63) |
| 12701 | Bringelly - Green Valley | 9.36(0.71) | 9.13(0.62) | 12.46(0.81) | 12.01(0.74) | 5.88(0.58) | 6.15(0.49) | 6.92(0.62) | 6.67(0.54) | 10.8(0.76) | 10.45(0.7) |
| 12702 | Fairfield | 9.84(0.6) | 10.4(0.57) | 13.12(0.68) | 13.35(0.66) | 6.46(0.5) | 7(0.45) | 9.6(0.59) | 10.09(0.56) | 15.21(0.72) | 15.35(0.71) |
| 12703 | Liverpool | 7.83(0.61) | 8.01(0.56) | 10.86(0.71) | 10.84(0.67) | 6.49(0.57) | 6.48(0.49) | 6.9(0.58) | 6.76(0.52) | 10.76(0.71) | 10.68(0.67) |
| 12801 | Cronulla - Miranda - Caringbah | 7.25(0.72) | 7.05(0.67) | 5.92(0.66) | 6.07(0.62) | 4.94(0.61) | 4.93(0.54) | 2.02(0.39) | 2.26(0.35) | 3.97(0.55) | 4.22(0.52) |
| 12802 | Sutherland - Menai - Heathcote | 5.35(0.6) | 5.43(0.5) | 6.19(0.64) | 6.26(0.56) | 5.33(0.6) | 5.14(0.48) | 2.6(0.42) | 2.55(0.31) | 3.94(0.52) | 4.17(0.46) |
| ***Victoria*** | | | | | | | | | | | |
| 20101 | Ballarat | 11.06(0.88) | 10.99(0.84) | 8.16(0.77) | 8.34(0.72) | 9.04(0.8) | 9.04(0.74) | 6.98(0.71) | 6.99(0.67) | 6.51(0.69) | 6.51(0.65) |
| 20102 | Creswick - Daylesford - Ballan | 10.47(1.91) | 9.5(1.05) | 7.66(1.65) | 8.34(1.03) | 7.66(1.65) | 8.29(0.89) | 4.26(1.26) | 5.81(0.75) | 3.49(1.14) | 5.43(0.81) |
| 20103 | Maryborough - Pyrenees | 14.4(2.19) | 13.33(1.37) | 13.62(2.14) | 12.41(1.43) | 9.16(1.82) | 10.28(1.12) | 10.89(1.94) | 10.41(1.22) | 7.78(1.67) | 8.5(1.16) |
| 20201 | Bendigo | 9.17(0.8) | 9.35(0.76) | 10.17(0.84) | 10.15(0.8) | 10.88(0.86) | 10.74(0.82) | 8.04(0.76) | 7.98(0.71) | 8.24(0.76) | 8.14(0.73) |
| 20202 | Heathcote - Castlemaine - Kyneton | 10(1.33) | 9.44(0.94) | 9.02(1.27) | 8.84(0.94) | 10.78(1.37) | 9.49(0.91) | 4.51(0.92) | 5.38(0.66) | 5.49(1.01) | 5.62(0.74) |
| 20203 | Loddon - Elmore | 11.4(2.98) | 11.18(1.44) | 7.89(2.53) | 10.16(1.44) | 7.02(2.39) | 9.61(1.2) | 10.53(2.87) | 8.81(1.28) | 9.65(2.77) | 8.2(1.34) |
| 20301 | Barwon - West | 6.75(1.58) | 7.66(1) | 4.37(1.29) | 6.6(0.95) | 6.77(1.59) | 7.66(0.99) | 6.35(1.54) | 5.24(0.81) | 5.16(1.39) | 5.16(0.86) |
| 20302 | Geelong | 8.88(0.61) | 8.99(0.58) | 9.35(0.63) | 9.38(0.59) | 10.68(0.67) | 10.38(0.62) | 6.4(0.53) | 6.55(0.5) | 7.03(0.55) | 7.1(0.53) |
| 20303 | Surf Coast - Bellarine Peninsula | 7.56(0.85) | 7.55(0.76) | 8.5(0.9) | 8.22(0.79) | 7.56(0.85) | 7.68(0.74) | 4.87(0.69) | 4.76(0.59) | 6.32(0.78) | 6(0.7) |
| 20401 | Upper Goulburn Valley | 6.88(1.08) | 8.53(0.78) | 7.79(1.14) | 8.67(0.87) | 8.51(1.19) | 8.82(0.77) | 7.43(1.12) | 7.44(0.74) | 3.44(0.78) | 5.33(0.68) |
| 20402 | Wangaratta - Benalla | 6.25(1.1) | 8.5(0.93) | 6.88(1.15) | 8.43(0.94) | 7.92(1.23) | 8.77(0.89) | 4.38(0.93) | 6.4(0.78) | 4.17(0.91) | 5.76(0.79) |
| 20403 | Wodonga - Alpine | 10.94(1.02) | 10.67(0.84) | 9.87(0.98) | 9.88(0.84) | 10.88(1.02) | 10.11(0.81) | 8.58(0.92) | 8.06(0.75) | 6.87(0.83) | 6.83(0.71) |
| 20501 | Baw Baw | 11.87(1.37) | 10.75(1.02) | 9.17(1.22) | 9.35(0.97) | 8.29(1.17) | 8.91(0.87) | 8.63(1.19) | 8.06(0.87) | 6.83(1.07) | 6.81(0.83) |
| 20502 | Gippsland - East | 12.76(1.53) | 12.26(1.2) | 10.88(1.42) | 10.79(1.13) | 8.82(1.3) | 9.33(0.97) | 7.95(1.24) | 8.4(0.97) | 7.53(1.21) | 7.65(0.97) |
| 20503 | Gippsland - South West | 9(1.07) | 9.56(0.93) | 10.66(1.15) | 10.65(0.99) | 9.57(1.1) | 9.84(0.91) | 7.07(0.95) | 7.58(0.83) | 8.45(1.04) | 8.27(0.9) |
| 20504 | Latrobe Valley | 16.41(1.23) | 15.74(1.12) | 16.52(1.23) | 15.77(1.12) | 15.1(1.19) | 14.22(1.07) | 12(1.08) | 11.89(0.99) | 13.22(1.12) | 12.74(1.05) |
| 20505 | Wellington | 10.47(1.32) | 10.58(0.98) | 9.35(1.26) | 9.82(0.99) | 10.28(1.31) | 10.06(0.93) | 10.65(1.33) | 9.49(0.97) | 7.48(1.14) | 7.51(0.88) |
| 20601 | Brunswick - Coburg | 5.64(0.85) | 5.89(0.62) | 7.52(0.97) | 7.45(0.75) | 7.58(0.97) | 7.22(0.68) | 2.82(0.61) | 3.58(0.45) | 3.49(0.67) | 4.5(0.59) |
| 20602 | Darebin - South | 3.6(0.79) | 4.85(0.65) | 4.68(0.9) | 5.66(0.74) | 5.06(0.95) | 5.9(0.72) | 4.32(0.86) | 3.92(0.58) | 3.78(0.81) | 4.29(0.66) |
| 20603 | Essendon | 4.12(0.74) | 4.92(0.61) | 6.17(0.89) | 6.65(0.75) | 7.02(0.95) | 6.95(0.74) | 3.02(0.63) | 3.42(0.5) | 4.12(0.74) | 4.73(0.65) |
| 20604 | Melbourne City | 7.46(1.08) | 7.07(0.74) | 9.15(1.19) | 8.65(0.89) | 8.14(1.13) | 7.82(0.76) | 5.08(0.9) | 4.8(0.59) | 11.02(1.29) | 9.21(1.01) |
| 20605 | Port Phillip | 4.62(0.76) | 4.98(0.56) | 5.28(0.81) | 5.72(0.63) | 7.29(0.95) | 6.73(0.67) | 2.24(0.54) | 2.75(0.38) | 3.03(0.62) | 3.87(0.53) |
| 20606 | Stonnington - West | 5.12(1.21) | 5.39(0.72) | 7.53(1.45) | 6.78(0.89) | 9.94(1.64) | 7.55(0.88) | 3.61(1.02) | 2.9(0.45) | 5.42(1.24) | 5.05(0.78) |
| 20607 | Yarra | 9.33(1.19) | 7.68(0.8) | 9.83(1.22) | 8.7(0.91) | 8.83(1.16) | 7.82(0.78) | 5.33(0.92) | 4.48(0.57) | 6.5(1.01) | 6.19(0.77) |
| 20701 | Boroondara | 4.73(0.52) | 4.88(0.43) | 4.61(0.52) | 5.03(0.46) | 4.92(0.53) | 5.35(0.44) | 1.82(0.33) | 2.21(0.26) | 4.73(0.52) | 4.64(0.45) |
| 20702 | Manningham - West | 6.59(0.82) | 6.25(0.64) | 10.91(1.02) | 9.61(0.86) | 7.34(0.86) | 6.86(0.66) | 4.97(0.71) | 4.28(0.52) | 8.53(0.92) | 7.46(0.76) |
| 20703 | Whitehorse - West | 4.48(0.64) | 5.11(0.54) | 5.25(0.69) | 5.89(0.6) | 5.34(0.69) | 5.84(0.56) | 2.67(0.5) | 3.2(0.41) | 4.68(0.65) | 5.06(0.58) |
| 20801 | Bayside | 3.53(0.55) | 3.88(0.47) | 4.33(0.6) | 4.65(0.53) | 4.9(0.64) | 5.07(0.55) | 2.56(0.47) | 2.48(0.37) | 3.18(0.52) | 3.33(0.45) |
| 20802 | Glen Eira | 4.94(0.56) | 5.07(0.45) | 6.53(0.63) | 6.42(0.54) | 5.69(0.6) | 5.88(0.48) | 1.72(0.33) | 2.35(0.28) | 4.48(0.53) | 4.54(0.45) |
| 20803 | Kingston | 5.48(0.61) | 5.49(0.53) | 5.9(0.63) | 6.05(0.56) | 4.21(0.54) | 4.85(0.48) | 3.7(0.5) | 3.6(0.41) | 4.05(0.53) | 4.25(0.47) |
| 20804 | Stonnington - East | 6.87(1.33) | 5.71(0.8) | 7.14(1.35) | 6.39(0.89) | 8.82(1.49) | 7.06(0.91) | 1.92(0.72) | 2.23(0.41) | 4.67(1.11) | 4.55(0.78) |
| 20901 | Banyule | 5.73(0.61) | 5.85(0.5) | 6.22(0.64) | 6.42(0.55) | 5.67(0.61) | 5.98(0.5) | 3.21(0.47) | 3.48(0.37) | 4.12(0.53) | 4.44(0.47) |
| 20902 | Darebin - North | 8.21(0.86) | 8.28(0.71) | 7.82(0.84) | 8.29(0.73) | 7.76(0.84) | 7.93(0.67) | 5.58(0.72) | 6.1(0.61) | 8.31(0.86) | 8.29(0.77) |
| 20903 | Nillumbik - Kinglake | 4.27(0.74) | 4.97(0.56) | 5.61(0.84) | 5.89(0.67) | 4.94(0.79) | 5.62(0.59) | 2.14(0.53) | 2.89(0.39) | 1.47(0.44) | 2.8(0.43) |
| 20904 | Whittlesea - Wallan | 8.14(0.49) | 8.19(0.45) | 7.79(0.48) | 7.95(0.46) | 6.8(0.45) | 7.05(0.42) | 6.47(0.44) | 6.48(0.4) | 7.37(0.47) | 7.34(0.44) |
| 21001 | Keilor | 5.84(0.96) | 6.08(0.67) | 8.68(1.15) | 8.25(0.87) | 5.18(0.91) | 6.24(0.67) | 3.85(0.79) | 4.14(0.53) | 5.51(0.93) | 5.8(0.73) |
| 21002 | Macedon Ranges | 8.53(1.34) | 7.5(0.82) | 7.37(1.25) | 7.04(0.84) | 6.91(1.22) | 6.93(0.74) | 4.38(0.98) | 4.09(0.55) | 4.15(0.96) | 4.35(0.64) |
| 21003 | Moreland - North | 9.23(0.93) | 8.99(0.78) | 12.21(1.05) | 11.53(0.91) | 9.37(0.94) | 8.94(0.76) | 9.03(0.92) | 8.31(0.76) | 12.21(1.05) | 11.41(0.94) |
| 21004 | Sunbury | 8.71(1.25) | 8.32(0.95) | 5.94(1.05) | 6.92(0.86) | 7.65(1.19) | 7.54(0.86) | 2.78(0.73) | 4.24(0.63) | 4.37(0.91) | 5.21(0.76) |
| 21005 | Tullamarine - Broadmeadows | 13.68(0.67) | 13.22(0.62) | 14.74(0.69) | 14.35(0.66) | 11.88(0.63) | 11.37(0.58) | 13.12(0.66) | 12.61(0.61) | 15.34(0.7) | 14.93(0.67) |
| 21101 | Knox | 6.38(0.61) | 6.37(0.52) | 6.76(0.63) | 6.81(0.56) | 7.16(0.65) | 7.05(0.55) | 4.36(0.51) | 4.41(0.43) | 5.37(0.57) | 5.46(0.51) |
| 21102 | Manningham - East | 6.77(1.59) | 5.53(0.74) | 6.77(1.59) | 6.15(0.87) | 7.57(1.67) | 6.27(0.79) | 3.59(1.17) | 3.16(0.51) | 4.38(1.29) | 4.14(0.7) |
| 21103 | Maroondah | 7.34(0.73) | 7.13(0.63) | 6.16(0.68) | 6.41(0.61) | 6.76(0.71) | 6.85(0.61) | 5.93(0.66) | 5.55(0.56) | 5.6(0.65) | 5.57(0.58) |
| 21104 | Whitehorse - East | 4.76(0.82) | 5.37(0.63) | 6.25(0.93) | 6.4(0.73) | 5.52(0.88) | 6.01(0.65) | 2.83(0.64) | 3.42(0.48) | 5.06(0.85) | 5.16(0.68) |
| 21105 | Yarra Ranges | 7.81(0.65) | 7.57(0.55) | 8.16(0.66) | 7.99(0.58) | 8.58(0.67) | 8.2(0.56) | 6.37(0.59) | 6(0.49) | 5.61(0.55) | 5.51(0.48) |
| 21201 | Cardinia | 7.5(0.66) | 7.64(0.57) | 8.8(0.71) | 8.73(0.64) | 9.37(0.73) | 9.09(0.63) | 6.08(0.6) | 6.17(0.53) | 4.47(0.51) | 4.88(0.48) |
| 21202 | Casey - North | 7.15(0.63) | 7.28(0.56) | 8.22(0.67) | 8.25(0.6) | 8.05(0.66) | 8.04(0.58) | 8(0.66) | 7.61(0.59) | 8.22(0.67) | 7.98(0.62) |
| 21203 | Casey - South | 8.19(0.48) | 8.15(0.45) | 8.35(0.48) | 8.4(0.46) | 7.59(0.46) | 7.66(0.43) | 6.76(0.44) | 6.75(0.41) | 8.13(0.47) | 8.03(0.46) |
| 21204 | Dandenong | 9.83(0.63) | 9.84(0.58) | 11.02(0.67) | 11.02(0.63) | 8.79(0.6) | 8.85(0.55) | 8.84(0.6) | 8.96(0.56) | 12.78(0.71) | 12.57(0.69) |
| 21205 | Monash | 6.05(0.54) | 6.02(0.46) | 7.04(0.58) | 6.99(0.52) | 6.29(0.56) | 6.38(0.47) | 3.6(0.43) | 3.55(0.34) | 6.68(0.57) | 6.47(0.51) |
| 21301 | Brimbank | 9.33(0.6) | 9.61(0.56) | 11.38(0.66) | 11.43(0.62) | 8.34(0.57) | 8.56(0.52) | 10.14(0.62) | 10.17(0.59) | 12.92(0.69) | 12.79(0.67) |
| 21302 | Hobsons Bay | 8.48(0.86) | 7.89(0.71) | 7.34(0.8) | 7.5(0.69) | 5.94(0.73) | 6.42(0.6) | 6.5(0.76) | 5.85(0.59) | 6.69(0.77) | 6.74(0.67) |
| 21303 | Maribyrnong | 5.57(0.75) | 6.19(0.63) | 7.16(0.84) | 7.59(0.73) | 6.56(0.81) | 6.95(0.64) | 3.32(0.59) | 4.24(0.49) | 6.94(0.83) | 7.05(0.72) |
| 21304 | Melton - Bacchus Marsh | 7.49(0.51) | 7.7(0.48) | 8.67(0.55) | 8.7(0.52) | 7.22(0.5) | 7.38(0.47) | 7.08(0.5) | 6.98(0.46) | 6.28(0.47) | 6.39(0.46) |
| 21305 | Wyndham | 8.94(0.43) | 8.86(0.41) | 10.57(0.46) | 10.41(0.45) | 8.27(0.41) | 8.21(0.4) | 7.63(0.4) | 7.47(0.38) | 9.9(0.45) | 9.69(0.44) |
| 21401 | Frankston | 7.66(0.66) | 7.67(0.59) | 9.06(0.71) | 8.94(0.65) | 8.37(0.68) | 8.11(0.6) | 5.9(0.58) | 5.93(0.52) | 6.09(0.59) | 6.21(0.55) |
| 21402 | Mornington Peninsula | 7.82(0.64) | 7.75(0.6) | 8.44(0.66) | 8.37(0.63) | 7.76(0.64) | 7.7(0.6) | 6.41(0.58) | 6.29(0.56) | 5.33(0.53) | 5.39(0.5) |
| 21501 | Grampians | 12.04(1.3) | 11.72(0.99) | 12.52(1.33) | 11.83(1.04) | 13.16(1.35) | 11.75(0.98) | 9.31(1.16) | 8.91(0.86) | 6.9(1.02) | 7.25(0.81) |
| 21502 | Mildura | 10.93(1.2) | 11.73(1.01) | 8.7(1.08) | 9.85(0.94) | 10.62(1.18) | 11.08(0.94) | 10.47(1.18) | 10.35(0.95) | 7.96(1.04) | 8.3(0.88) |
| 21503 | Murray River - Swan Hill | 9.95(1.42) | 10.74(1.09) | 12.02(1.55) | 11.49(1.17) | 7.97(1.29) | 9.06(0.94) | 7.31(1.24) | 8.12(0.95) | 7.01(1.21) | 7.41(0.95) |
| 21601 | Campaspe | 11.62(1.5) | 11.32(1.07) | 12.5(1.55) | 11.63(1.15) | 9.47(1.37) | 9.46(0.93) | 8.79(1.33) | 8.43(0.92) | 7.89(1.26) | 7.86(0.96) |
| 21602 | Moira | 10.51(1.73) | 11.17(1.31) | 9.24(1.63) | 10.25(1.28) | 6.89(1.45) | 8.61(1.06) | 6.69(1.41) | 7.9(1.09) | 8.28(1.56) | 8.53(1.23) |
| 21603 | Shepparton | 16.61(1.29) | 15.27(1.13) | 15.53(1.25) | 14.47(1.11) | 14.58(1.22) | 13.12(1.03) | 13.62(1.19) | 12.47(1.05) | 15.05(1.24) | 13.73(1.12) |
| 21701 | Glenelg - Southern Grampians | 13.76(1.83) | 12.66(1.44) | 13.48(1.81) | 12.31(1.42) | 14.61(1.87) | 12.86(1.41) | 6.74(1.33) | 7.37(1.07) | 10.96(1.66) | 9.56(1.31) |
| 21703 | Colac - Corangamite | 11.57(1.49) | 10.98(1.13) | 10.92(1.46) | 10.55(1.14) | 10.04(1.4) | 9.74(1) | 6.55(1.16) | 7.33(0.91) | 7.21(1.21) | 7.23(0.95) |
| 21704 | Warrnambool | 6.04(1.03) | 7.5(0.93) | 6.42(1.06) | 7.58(0.93) | 5.47(0.99) | 7.33(0.89) | 7.36(1.13) | 7.03(0.91) | 4.15(0.87) | 4.98(0.78) |
| ***Queensland*** | | | | | | | | | | | |
| 30101 | Capalaba | 14.4(1.21) | 13.38(1.05) | 13.59(1.18) | 12.88(1.05) | 11.93(1.12) | 11.15(0.94) | 8.7(0.97) | 7.47(0.78) | 10.83(1.07) | 10.22(0.95) |
| 30102 | Cleveland - Stradbroke | 7.04(0.84) | 8.48(0.77) | 7.69(0.88) | 8.73(0.8) | 7.48(0.87) | 8.56(0.75) | 5.09(0.72) | 5.74(0.62) | 4.55(0.69) | 5.81(0.67) |
| 30103 | Wynnum - Manly | 10.08(0.98) | 10.43(0.91) | 11.68(1.05) | 11.53(0.97) | 9.48(0.97) | 9.38(0.84) | 3.72(0.62) | 4.17(0.55) | 6.37(0.8) | 6.77(0.75) |
| 30201 | Bald Hills - Everton Park | 7.49(1.15) | 8.33(0.87) | 9.98(1.31) | 9.8(1.02) | 9.6(1.29) | 9.36(0.92) | 3.65(0.82) | 4.19(0.56) | 8.25(1.21) | 7.82(0.94) |
| 30202 | Chermside | 9.08(1) | 9.17(0.84) | 9.58(1.02) | 9.66(0.87) | 9.94(1.04) | 9.56(0.83) | 4.13(0.69) | 4.48(0.55) | 7.87(0.94) | 7.9(0.82) |
| 30203 | Nundah | 10.85(1.51) | 10.47(1.23) | 13.44(1.66) | 12.34(1.34) | 9.2(1.4) | 9.22(1.09) | 6.37(1.19) | 5.87(0.88) | 8.25(1.34) | 8.33(1.14) |
| 30204 | Sandgate | 10.74(1.09) | 10.83(0.95) | 11.85(1.14) | 11.94(1.01) | 8.81(1) | 9.38(0.86) | 6.68(0.88) | 6.64(0.74) | 10.49(1.08) | 10.41(0.97) |
| 30301 | Carindale | 10.03(1.19) | 9.84(0.95) | 10.03(1.19) | 9.78(0.98) | 8.63(1.11) | 8.5(0.84) | 3.13(0.69) | 3.6(0.5) | 6.11(0.95) | 6.46(0.8) |
| 30302 | Holland Park - Yeronga | 6.88(0.95) | 8.03(0.77) | 7.3(0.98) | 8.06(0.8) | 7.07(0.96) | 7.66(0.72) | 3.79(0.72) | 4(0.5) | 6.6(0.93) | 7.01(0.78) |
| 30303 | Mt Gravatt | 10.44(1.02) | 10.33(0.83) | 11.44(1.06) | 11.07(0.9) | 11.37(1.06) | 10.46(0.82) | 5.9(0.79) | 5.59(0.59) | 12.11(1.09) | 11.11(0.92) |
| 30304 | Nathan | 10.99(1.47) | 10.44(1.13) | 9.47(1.37) | 9.81(1.11) | 8.59(1.32) | 8.81(0.99) | 3.52(0.86) | 4.55(0.68) | 5.93(1.11) | 7.26(0.99) |
| 30305 | Rocklea - Acacia Ridge | 10.81(1) | 10.65(0.82) | 13.1(1.09) | 12.33(0.94) | 9.81(0.96) | 9.57(0.76) | 8.12(0.88) | 7.07(0.68) | 11.75(1.04) | 11.14(0.91) |
| 30306 | Sunnybank | 9.69(1.11) | 10.27(0.97) | 11.81(1.21) | 11.82(1.05) | 8.62(1.05) | 9.23(0.89) | 6.88(0.95) | 6.85(0.79) | 16.01(1.37) | 14.99(1.24) |
| 30401 | Centenary | 16.47(1.79) | 15.29(1.62) | 10.9(1.5) | 10.46(1.36) | 13.23(1.63) | 12.25(1.46) | 3.72(0.91) | 3.9(0.8) | 5.8(1.13) | 5.94(1.03) |
| 30402 | Kenmore - Brookfield - Moggill | 9.44(1.19) | 8.76(0.91) | 10.93(1.27) | 9.59(0.99) | 11.92(1.32) | 9.98(0.97) | 2.32(0.61) | 2.84(0.42) | 5.46(0.92) | 5.35(0.71) |
| 30403 | Sherwood - Indooroopilly | 6.22(1) | 7.22(0.78) | 3.11(0.72) | 5.68(0.71) | 5.7(0.96) | 7.02(0.73) | 1.38(0.49) | 3.03(0.44) | 5.18(0.92) | 5.78(0.74) |
| 30404 | The Gap - Enoggera | 7.32(0.99) | 7.93(0.74) | 5.31(0.85) | 6.77(0.72) | 6.33(0.92) | 7.47(0.7) | 2.61(0.61) | 3.5(0.43) | 5.6(0.87) | 5.95(0.69) |
| 30501 | Brisbane Inner | 8.17(1.43) | 8.72(1.09) | 9.26(1.51) | 9.32(1.16) | 7.65(1.39) | 7.85(0.97) | 2.72(0.85) | 3.83(0.64) | 9.54(1.53) | 8.81(1.2) |
| 30502 | Brisbane Inner - East | 11.33(1.36) | 10.16(1.07) | 8.23(1.17) | 8.34(0.95) | 5.14(0.95) | 6.35(0.76) | 2.93(0.72) | 3.03(0.48) | 6.95(1.09) | 6.64(0.87) |
| 30503 | Brisbane Inner - North | 8.97(0.96) | 8.6(0.78) | 7.49(0.89) | 7.81(0.76) | 8.85(0.96) | 8.42(0.77) | 4.77(0.72) | 4.22(0.53) | 7.15(0.87) | 6.91(0.75) |
| 30504 | Brisbane Inner - West | 5.06(0.87) | 5.91(0.73) | 7.58(1.05) | 7.35(0.84) | 5.54(0.91) | 6.26(0.72) | 2.7(0.65) | 2.8(0.45) | 3.48(0.73) | 4.27(0.63) |
| 30601 | Cairns - North | 10.33(1.18) | 10.35(1.03) | 6.74(0.97) | 7.35(0.86) | 7.63(1.03) | 7.98(0.88) | 4.22(0.78) | 4.79(0.67) | 5.09(0.85) | 5.84(0.78) |
| 30602 | Cairns - South | 12.06(0.87) | 12.23(0.83) | 12.13(0.87) | 12.03(0.82) | 11.26(0.85) | 11(0.78) | 9.36(0.78) | 9.32(0.73) | 11(0.84) | 10.99(0.8) |
| 30603 | Innisfail - Cassowary Coast | 12.95(1.6) | 13.74(1.39) | 10.91(1.49) | 11.97(1.31) | 7.95(1.29) | 9.49(1.11) | 7.97(1.29) | 9.7(1.19) | 11.59(1.53) | 12.26(1.39) |
| 30604 | Port Douglas - Daintree | 14.73(3.12) | 13.68(2.01) | 9.3(2.56) | 10.15(1.67) | 9.3(2.56) | 9.82(1.52) | 7.75(2.35) | 8.25(1.49) | 17.83(3.37) | 13.71(2.21) |
| 30605 | Tablelands (East) - Kuranda | 17.02(1.72) | 15.64(1.33) | 15.13(1.64) | 13.88(1.31) | 11.34(1.45) | 10.91(1.05) | 14.32(1.62) | 12.36(1.2) | 14.5(1.61) | 13.63(1.34) |
| 30701 | Darling Downs (West) - Maranoa | 16.19(1.43) | 14.73(1.09) | 11.35(1.23) | 11.48(1) | 9.47(1.14) | 9.81(0.82) | 10.14(1.17) | 9.55(0.86) | 12.1(1.27) | 11.35(1.03) |
| 30702 | Darling Downs - East | 12.26(1.38) | 12.83(1.13) | 10.48(1.29) | 10.98(1.04) | 9.24(1.22) | 9.77(0.91) | 11.01(1.32) | 10.1(0.99) | 10.48(1.29) | 10.37(1.07) |
| 30703 | Granite Belt | 14.86(1.59) | 14.69(1.29) | 15.06(1.6) | 14.16(1.29) | 11.29(1.42) | 10.93(1.05) | 9.84(1.33) | 9.92(1.06) | 12.25(1.47) | 11.91(1.24) |
| 30801 | Central Highlands (Qld) | 8.92(1.31) | 10.32(1.08) | 14.65(1.63) | 13.41(1.29) | 11.46(1.47) | 10.61(1.05) | 9.34(1.34) | 9.09(1.03) | 7.22(1.19) | 8.07(1) |
| 30803 | Rockhampton | 14.67(0.9) | 14.28(0.84) | 14.09(0.88) | 13.85(0.84) | 10.7(0.78) | 10.64(0.71) | 9.72(0.75) | 9.8(0.7) | 11.65(0.81) | 11.46(0.77) |
| 30804 | Biloela | 11.11(2.29) | 11.44(1.44) | 10.05(2.19) | 10.67(1.43) | 8.47(2.02) | 9.53(1.2) | 8.47(2.02) | 8.18(1.16) | 10.05(2.19) | 9.29(1.41) |
| 30805 | Gladstone | 11.05(1.04) | 11.49(0.94) | 10.5(1.01) | 10.79(0.93) | 11.07(1.04) | 10.95(0.88) | 10.18(1) | 9.93(0.89) | 8.98(0.95) | 9.19(0.87) |
| 30901 | Broadbeach - Burleigh | 6.26(1.02) | 6.99(0.8) | 11.81(1.36) | 10.51(1.07) | 8.3(1.17) | 7.93(0.85) | 5.01(0.92) | 4.7(0.63) | 5.9(1) | 6.11(0.82) |
| 30902 | Coolangatta | 6.03(0.99) | 6.71(0.85) | 7.07(1.06) | 7.79(0.93) | 5.54(0.95) | 6.38(0.79) | 3.45(0.76) | 3.98(0.62) | 6.03(0.99) | 6.13(0.85) |
| 30903 | Gold Coast - North | 8.95(1.13) | 9.44(1) | 10.68(1.22) | 10.83(1.07) | 9.34(1.16) | 9.45(0.97) | 8.98(1.13) | 8.58(0.96) | 10.83(1.23) | 10.72(1.11) |
| 30904 | Gold Coast Hinterland | 10(2.02) | 9.48(1.1) | 9.09(1.94) | 9.3(1.17) | 8.18(1.85) | 8.22(0.94) | 4.09(1.34) | 4.68(0.66) | 8.64(1.89) | 7.38(1.09) |
| 30905 | Mudgeeraba - Tallebudgera | 7.98(1.2) | 7.56(0.84) | 8.37(1.22) | 8.19(0.92) | 6.25(1.07) | 6.82(0.74) | 3.5(0.81) | 3.66(0.52) | 4.47(0.91) | 4.96(0.7) |
| 30906 | Nerang | 9.28(0.98) | 9.17(0.77) | 10.07(1.01) | 9.95(0.85) | 10.56(1.04) | 9.59(0.77) | 5.43(0.76) | 5.61(0.57) | 8.03(0.91) | 8.01(0.76) |
| 30907 | Ormeau - Oxenford | 11.14(0.67) | 10.95(0.61) | 12.57(0.7) | 12.2(0.65) | 10.32(0.64) | 10.09(0.59) | 8.15(0.58) | 7.64(0.51) | 9.13(0.61) | 8.92(0.58) |
| 30908 | Robina | 9.27(1.21) | 8.87(0.99) | 7.69(1.11) | 8.21(0.96) | 9.17(1.21) | 8.76(0.98) | 4.72(0.89) | 4.74(0.71) | 5.07(0.92) | 5.52(0.8) |
| 30909 | Southport | 10.19(1.31) | 10.07(1.12) | 9.43(1.27) | 9.81(1.09) | 7.58(1.15) | 8.2(0.96) | 5.85(1.02) | 6.44(0.87) | 11.32(1.38) | 11.14(1.24) |
| 30910 | Surfers Paradise | 8.3(1.66) | 8.49(1.23) | 7.58(1.59) | 8.67(1.25) | 6.16(1.45) | 7.45(1.08) | 6.86(1.52) | 6.15(1.03) | 11.19(1.89) | 10.02(1.47) |
| 31001 | Forest Lake - Oxley | 13.25(1.01) | 13.89(0.94) | 14.31(1.04) | 14.44(0.96) | 11.22(0.94) | 11.62(0.83) | 12.02(0.97) | 11.72(0.88) | 14.75(1.05) | 14.72(0.98) |
| 31002 | Ipswich Hinterland | 14.72(1.23) | 14.4(0.96) | 11.7(1.12) | 12.11(0.92) | 10.58(1.07) | 10.72(0.79) | 9.66(1.03) | 9.34(0.75) | 8.09(0.95) | 9.16(0.83) |
| 31003 | Ipswich Inner | 14.88(0.88) | 14.87(0.84) | 16.24(0.91) | 16(0.87) | 12.43(0.82) | 12.4(0.76) | 10.65(0.77) | 10.44(0.71) | 13.92(0.86) | 13.73(0.83) |
| 31004 | Springfield - Redbank | 14.53(0.83) | 14.43(0.77) | 14.21(0.82) | 14.15(0.78) | 12.55(0.78) | 12.39(0.71) | 8.25(0.65) | 8.32(0.6) | 11.82(0.76) | 11.81(0.73) |
| 31101 | Beaudesert | 14.29(2.7) | 14.85(1.84) | 17.86(2.95) | 15.77(1.94) | 12.5(2.55) | 11.61(1.49) | 12.5(2.55) | 11.02(1.59) | 13.1(2.6) | 12.74(1.86) |
| 31102 | Beenleigh | 17.77(1.57) | 17.66(1.42) | 12.35(1.35) | 13.25(1.23) | 12.01(1.34) | 12.43(1.16) | 11.02(1.29) | 11.62(1.17) | 10.51(1.26) | 11.32(1.19) |
| 31103 | Browns Plains | 15.59(0.97) | 15.46(0.87) | 14.52(0.94) | 14.53(0.87) | 11.19(0.84) | 11.44(0.75) | 9.91(0.8) | 10.01(0.72) | 14.66(0.94) | 14.51(0.89) |
| 31104 | Jimboomba | 12.53(1.15) | 12.1(0.9) | 14.23(1.22) | 13.15(1) | 11.19(1.1) | 10.54(0.81) | 8.88(0.99) | 7.56(0.7) | 10.1(1.05) | 9.46(0.86) |
| 31105 | Loganlea - Carbrook | 16.78(1.25) | 15.73(1.07) | 15.54(1.22) | 14.77(1.05) | 13.69(1.16) | 12.7(0.93) | 8.91(0.96) | 8.73(0.79) | 12.39(1.11) | 11.89(0.99) |
| 31106 | Springwood - Kingston | 16.78(1.08) | 16.48(0.97) | 15.35(1.04) | 15.4(0.95) | 13.94(1.01) | 13.54(0.87) | 11.66(0.93) | 11.72(0.83) | 14.09(1.01) | 14.39(0.95) |
| 31201 | Bowen Basin - North | 8.3(1.24) | 10.07(1.02) | 8.92(1.28) | 10.28(1.09) | 5.69(1.04) | 8.3(0.86) | 8.5(1.25) | 8.86(0.96) | 7.09(1.15) | 8.06(0.97) |
| 31202 | Mackay | 12.03(0.82) | 11.92(0.79) | 11.2(0.8) | 11.28(0.78) | 10.51(0.78) | 10.45(0.74) | 8.45(0.7) | 8.45(0.68) | 11.2(0.8) | 10.96(0.77) |
| 31203 | Whitsunday | 11.48(2.04) | 11.16(1.59) | 15.57(2.32) | 13.77(1.81) | 13.52(2.19) | 11.62(1.59) | 7.79(1.72) | 7.9(1.34) | 6.15(1.54) | 7.17(1.32) |
| 31301 | Bribie - Beachmere | 11.62(1.9) | 12.17(1.62) | 12.32(1.95) | 12.61(1.63) | 12.86(2) | 12.72(1.62) | 11.97(1.93) | 10.93(1.57) | 9.15(1.71) | 9.39(1.47) |
| 31302 | Caboolture | 15.62(1.15) | 15.42(1.06) | 15.72(1.15) | 15.33(1.05) | 16.7(1.18) | 15.57(1.05) | 10.05(0.95) | 10.22(0.87) | 11.74(1.02) | 11.68(0.94) |
| 31303 | Caboolture Hinterland | 12.58(2.63) | 13.12(1.4) | 5.66(1.83) | 10.2(1.31) | 3.77(1.51) | 9.52(1.07) | 5.66(1.83) | 8.05(1.03) | 4.4(1.63) | 8.28(1.22) |
| 31304 | Narangba - Burpengary | 14.43(1.19) | 13.94(1.04) | 10.28(1.03) | 10.72(0.91) | 9.1(0.98) | 9.8(0.83) | 7.97(0.92) | 7.88(0.78) | 11.09(1.07) | 10.75(0.95) |
| 31305 | Redcliffe | 12.34(1.33) | 12.78(1.22) | 12.5(1.33) | 12.77(1.22) | 12.25(1.33) | 12.19(1.18) | 8.44(1.12) | 8.76(1.02) | 9.25(1.17) | 9.78(1.1) |
| 31401 | The Hills District | 8.65(0.81) | 8.68(0.69) | 8.32(0.8) | 8.34(0.69) | 7.5(0.76) | 7.84(0.62) | 4.45(0.6) | 4.19(0.44) | 6.16(0.69) | 6.07(0.59) |
| 31402 | North Lakes | 12.5(0.88) | 12.24(0.79) | 15.16(0.95) | 14.5(0.87) | 12.24(0.87) | 11.77(0.76) | 7.79(0.71) | 7.44(0.62) | 11.24(0.84) | 10.9(0.77) |
| 31403 | Strathpine | 15.77(1.57) | 14.91(1.35) | 17.07(1.62) | 16.09(1.42) | 16.33(1.59) | 14.83(1.34) | 10.41(1.32) | 9.7(1.1) | 12.62(1.43) | 12.33(1.27) |
| 31501 | Far North | 20(1.78) | 20.64(1.59) | 18.06(1.71) | 18.38(1.51) | 16.3(1.66) | 15.9(1.38) | 22.47(1.86) | 22.86(1.69) | 21.63(1.83) | 21.95(1.69) |
| 31502 | Outback - North | 18.16(1.72) | 17.41(1.42) | 15.17(1.6) | 14.84(1.33) | 14.6(1.58) | 13.76(1.22) | 16.2(1.65) | 15.52(1.39) | 14.77(1.59) | 14.36(1.37) |
| 31503 | Outback - South | 19.67(2.54) | 15.37(1.42) | 12.3(2.1) | 12.3(1.32) | 13.03(2.18) | 11.5(1.11) | 15.16(2.3) | 12.35(1.25) | 17.62(2.44) | 13.25(1.56) |
| 31601 | Buderim | 6.64(0.99) | 7.41(0.84) | 8.53(1.11) | 8.94(0.95) | 7.27(1.03) | 8.07(0.86) | 3.79(0.76) | 4.33(0.61) | 5.69(0.92) | 5.96(0.78) |
| 31602 | Caloundra | 9.76(0.96) | 9.66(0.88) | 11.65(1.04) | 11.46(0.94) | 9.91(0.97) | 9.84(0.86) | 5.77(0.76) | 5.78(0.67) | 7.45(0.85) | 7.42(0.78) |
| 31603 | Maroochy | 10.45(1.28) | 9.71(1.02) | 14.63(1.48) | 13.22(1.22) | 12.78(1.4) | 11.31(1.09) | 7.32(1.09) | 6.62(0.83) | 8.89(1.19) | 8.43(0.99) |
| 31605 | Noosa | 3.93(0.91) | 6.02(0.85) | 4.8(1) | 6.83(0.93) | 4.8(1) | 7.04(0.91) | 4.15(0.93) | 4.77(0.75) | 5.24(1.04) | 6.01(0.91) |
| 31606 | Sunshine Coast Hinterland | 9.91(1.28) | 9.94(0.92) | 9.91(1.28) | 10.03(0.99) | 7.71(1.14) | 9(0.86) | 5.14(0.95) | 5.58(0.65) | 5.69(0.99) | 6.41(0.79) |
| 31607 | Nambour | 10.21(1.27) | 9.92(1) | 11.09(1.32) | 10.98(1.08) | 10.74(1.3) | 10.35(1) | 6.87(1.06) | 6.69(0.81) | 9.51(1.23) | 9.09(1.02) |
| 31608 | Noosa Hinterland | 12.4(2.12) | 10.49(1.37) | 12.4(2.12) | 10.79(1.43) | 14.46(2.26) | 11.19(1.36) | 7.02(1.64) | 6.13(0.97) | 11.16(2.02) | 9.09(1.38) |
| 31701 | Toowoomba | 13.4(0.75) | 13.31(0.72) | 11.39(0.7) | 11.35(0.66) | 10.39(0.67) | 10.29(0.63) | 8.33(0.6) | 8.26(0.57) | 10.29(0.66) | 10.17(0.64) |
| 31801 | Charters Towers - Ayr - Ingham | 12.73(1.5) | 13.85(1.18) | 13.54(1.54) | 13.62(1.23) | 10.1(1.35) | 10.86(0.99) | 11.72(1.45) | 12.3(1.14) | 11.52(1.43) | 12.13(1.22) |
| 31802 | Townsville | 14.91(0.71) | 14.81(0.71) | 13.59(0.69) | 13.51(0.68) | 11.06(0.63) | 10.99(0.62) | 10.96(0.62) | 10.89(0.63) | 11.27(0.63) | 11.23(0.64) |
| 31901 | Bundaberg | 17.77(1.17) | 17.3(1.1) | 15.72(1.11) | 15.27(1.03) | 13.98(1.06) | 13.57(0.96) | 14.89(1.09) | 14.31(1) | 14.7(1.08) | 14.36(1.03) |
| 31902 | Burnett | 15.69(1.51) | 16.06(1.22) | 13.62(1.42) | 13.94(1.18) | 13.02(1.4) | 12.76(1.04) | 11.55(1.33) | 12.53(1.09) | 13.62(1.42) | 13.71(1.2) |
| 31903 | Gympie - Cooloola | 14.75(1.51) | 14.69(1.26) | 12.2(1.4) | 12.56(1.19) | 12.02(1.39) | 11.99(1.09) | 9.84(1.27) | 10.01(1.04) | 9.84(1.27) | 10.36(1.12) |
| 31904 | Hervey Bay | 26.7(1.82) | 25.79(1.77) | 17.86(1.58) | 17.24(1.49) | 13.78(1.42) | 13.5(1.32) | 10.37(1.26) | 10.18(1.17) | 16.84(1.54) | 16.34(1.48) |
| 31905 | Maryborough | 14.58(1.54) | 15.86(1.37) | 11.17(1.37) | 12.49(1.23) | 11.74(1.4) | 12.41(1.18) | 9.28(1.26) | 10.51(1.11) | 10.42(1.33) | 11.49(1.21) |
| ***South Australia*** | | | | | | | | | | | |
| 40101 | Adelaide City | 12.37(3.34) | 8.31(1.2) | 17.53(3.86) | 10.77(1.6) | 12.37(3.34) | 8.83(1.19) | 9.28(2.95) | 5.36(0.89) | 13.4(3.46) | 8.1(1.41) |
| 40102 | Adelaide Hills | 10.81(1.08) | 9.46(0.79) | 10.69(1.08) | 9.8(0.85) | 11.06(1.09) | 10.15(0.8) | 4.71(0.75) | 4.33(0.47) | 6.08(0.83) | 5.72(0.63) |
| 40103 | Burnside | 5.08(0.95) | 5.83(0.7) | 8.27(1.19) | 7.93(0.89) | 9.7(1.29) | 8.62(0.87) | 3.57(0.8) | 3.46(0.49) | 6.2(1.05) | 5.56(0.75) |
| 40104 | Campbelltown (SA) | 7.1(1.11) | 7.78(0.86) | 8.97(1.24) | 9.18(0.99) | 7.16(1.12) | 8.24(0.87) | 4.32(0.88) | 4.92(0.66) | 7.1(1.11) | 7.18(0.89) |
| 40105 | Norwood - Payneham - St Peters | 6.14(1.3) | 6.95(0.9) | 6.14(1.3) | 7.56(0.99) | 8.43(1.53) | 8.4(1) | 7.33(1.41) | 5.54(0.81) | 9.06(1.55) | 7.87(1.1) |
| 40106 | Prospect - Walkerville | 5.72(1.35) | 6.48(0.89) | 4.38(1.19) | 6.52(0.93) | 6.44(1.43) | 7.24(0.93) | 2.69(0.94) | 3.54(0.58) | 3.7(1.1) | 4.91(0.82) |
| 40107 | Unley | 4.2(1) | 5.66(0.75) | 7.16(1.28) | 7.56(0.94) | 5.69(1.15) | 6.92(0.82) | 3.46(0.91) | 3.36(0.53) | 2.47(0.77) | 3.92(0.66) |
| 40201 | Gawler - Two Wells | 8.04(1.32) | 8.68(1.14) | 9.22(1.41) | 9.84(1.23) | 8.75(1.37) | 9.77(1.2) | 5.91(1.15) | 6.33(0.99) | 7.57(1.29) | 7.51(1.12) |
| 40202 | Playford | 16.1(0.95) | 16(0.89) | 16.42(0.96) | 16.45(0.9) | 15.79(0.94) | 15.54(0.86) | 14.92(0.92) | 14.82(0.87) | 14.83(0.92) | 14.79(0.88) |
| 40203 | Port Adelaide - East | 11.34(1.11) | 10.87(0.9) | 11.71(1.13) | 11.55(0.97) | 9.67(1.04) | 9.75(0.83) | 7.41(0.92) | 7.35(0.73) | 10.23(1.06) | 10.01(0.94) |
| 40204 | Salisbury | 12.27(0.76) | 12.24(0.72) | 13.63(0.8) | 13.55(0.77) | 11.15(0.73) | 11.18(0.69) | 8.51(0.65) | 8.56(0.61) | 11.68(0.75) | 11.6(0.71) |
| 40205 | Tea Tree Gully | 8.12(0.84) | 8.22(0.71) | 8.98(0.88) | 9.02(0.77) | 8.42(0.85) | 8.62(0.73) | 4.64(0.65) | 4.63(0.52) | 5.76(0.72) | 5.92(0.63) |
| 40301 | Holdfast Bay | 8.9(1.67) | 8.32(1.28) | 10.62(1.8) | 9.64(1.39) | 9.59(1.72) | 8.49(1.24) | 2.75(0.96) | 3.02(0.68) | 5.14(1.29) | 5.16(1.01) |
| 40302 | Marion | 8.39(0.87) | 8.51(0.76) | 9.88(0.94) | 9.9(0.82) | 9.15(0.91) | 9.11(0.76) | 4.3(0.64) | 4.45(0.52) | 7.12(0.81) | 6.89(0.7) |
| 40303 | Mitcham | 6.51(0.94) | 6.74(0.73) | 8.39(1.05) | 8.31(0.85) | 9.14(1.1) | 8.85(0.85) | 3.48(0.7) | 3.49(0.49) | 4.78(0.81) | 4.79(0.65) |
| 40304 | Onkaparinga | 10.89(0.69) | 10.87(0.65) | 11.33(0.7) | 11.25(0.66) | 11.51(0.71) | 11.4(0.65) | 6(0.53) | 6.01(0.49) | 6.4(0.54) | 6.56(0.52) |
| 40401 | Charles Sturt | 8.45(0.86) | 8.54(0.76) | 10.85(0.96) | 10.64(0.86) | 8.9(0.89) | 8.77(0.76) | 6.01(0.74) | 5.79(0.63) | 8.08(0.84) | 7.95(0.76) |
| 40402 | Port Adelaide - West | 10.71(1.26) | 10.9(1.05) | 12.19(1.33) | 12.27(1.16) | 9.78(1.21) | 10.04(0.98) | 6.77(1.02) | 7.41(0.87) | 8.9(1.16) | 9.4(1.05) |
| 40403 | West Torrens | 8.56(1.18) | 8.39(0.89) | 6.94(1.07) | 8.08(0.9) | 4.46(0.87) | 6.59(0.75) | 2.5(0.66) | 3.72(0.54) | 4.8(0.9) | 5.53(0.74) |
| 40501 | Barossa | 8.87(1.41) | 9.36(1.06) | 13.05(1.67) | 11.67(1.25) | 14.78(1.76) | 12.61(1.24) | 7.67(1.32) | 6.74(0.88) | 4.68(1.05) | 5.73(0.84) |
| 40502 | Lower North | 11.19(1.89) | 11.22(1.32) | 9.03(1.72) | 10.43(1.3) | 7.94(1.62) | 10.44(1.21) | 8.3(1.66) | 8.09(1.12) | 9.39(1.75) | 8.62(1.25) |
| 40503 | Mid North | 11.76(1.84) | 12.68(1.62) | 13.4(1.95) | 13.64(1.65) | 14.14(2) | 13.89(1.64) | 10.13(1.72) | 10.93(1.53) | 9.15(1.65) | 9.79(1.47) |
| 40504 | Yorke Peninsula | 8.64(1.89) | 9.59(1.72) | 12.27(2.21) | 12.23(1.89) | 11.36(2.14) | 11.44(1.81) | 5.45(1.53) | 6.64(1.42) | 7.27(1.75) | 7.85(1.57) |
| 40601 | Eyre Peninsula and South West | 15.11(1.36) | 15.02(1.27) | 15.71(1.38) | 15.13(1.27) | 12.27(1.25) | 12.18(1.12) | 10.37(1.16) | 10.77(1.1) | 11.1(1.19) | 11.02(1.12) |
| 40602 | Outback - North and East | 22.06(2.25) | 18.48(1.47) | 24.48(2.34) | 19.61(1.82) | 21.89(2.25) | 16.32(1.38) | 19.76(2.16) | 17.18(1.44) | 15.34(1.96) | 14.06(1.42) |
| 40701 | Fleurieu - Kangaroo Island | 15.35(1.66) | 14.37(1.41) | 10.66(1.43) | 10.82(1.21) | 11.73(1.49) | 11.72(1.21) | 8.33(1.28) | 7.83(1.01) | 10.45(1.41) | 9.73(1.19) |
| 40702 | Limestone Coast | 13.53(1.21) | 13.19(1.07) | 12.03(1.15) | 11.79(1) | 12.91(1.19) | 12.56(1.02) | 8.77(1) | 8.67(0.86) | 8.27(0.98) | 8.25(0.87) |
| 40703 | Murray and Mallee | 14.54(1.29) | 14.46(1.09) | 10.9(1.14) | 11.65(0.99) | 12.7(1.22) | 12.9(0.99) | 8.77(1.04) | 9.68(0.87) | 9.42(1.07) | 9.75(0.94) |
| ***Western Australia*** | | | | | | | | | | | |
| 50101 | Augusta - Margaret River - Busselton | 5.76(0.85) | 6.54(0.8) | 5.63(0.84) | 5.88(0.75) | 7.34(0.96) | 7.59(0.86) | 4.77(0.79) | 5.17(0.73) | 5.35(0.82) | 5.37(0.75) |
| 50102 | Bunbury | 12.77(0.91) | 12.13(0.83) | 8.46(0.76) | 8.19(0.69) | 10.27(0.83) | 9.79(0.73) | 8.32(0.75) | 8.08(0.68) | 7.72(0.73) | 7.53(0.66) |
| 50103 | Manjimup | 9.21(1.92) | 9.87(1.32) | 5.7(1.54) | 6.92(1.06) | 8.33(1.83) | 8.68(1.15) | 8.77(1.87) | 8.29(1.22) | 3.95(1.29) | 5.78(1.04) |
| 50201 | Mandurah | 9.76(0.85) | 9.98(0.77) | 7.17(0.74) | 7.48(0.67) | 8.33(0.79) | 8.42(0.7) | 7.17(0.74) | 7.31(0.66) | 8.63(0.8) | 8.47(0.74) |
| 50301 | Cottesloe - Claremont | 4.08(0.7) | 4.49(0.59) | 3.34(0.63) | 3.78(0.53) | 4.21(0.71) | 4.61(0.58) | 2.1(0.5) | 2.39(0.4) | 2.23(0.52) | 2.65(0.45) |
| 50302 | Perth City | 5.64(0.75) | 5.71(0.65) | 4.79(0.7) | 4.87(0.59) | 5.88(0.77) | 5.77(0.64) | 2.87(0.54) | 3.19(0.47) | 2.98(0.55) | 3.27(0.49) |
| 50401 | Bayswater - Bassendean | 8.61(0.94) | 8.27(0.82) | 6.46(0.83) | 6.42(0.72) | 6.69(0.84) | 6.72(0.71) | 6.46(0.83) | 6.15(0.71) | 4.88(0.73) | 5.12(0.64) |
| 50402 | Mundaring | 7.73(1.25) | 8.13(1.02) | 8.17(1.29) | 7.64(1) | 9.29(1.37) | 8.61(1.04) | 7.1(1.21) | 6.48(0.91) | 8.61(1.32) | 8.03(1.09) |
| 50403 | Swan | 8.41(0.64) | 8.47(0.56) | 7.3(0.6) | 7.32(0.54) | 7.79(0.61) | 7.76(0.53) | 6.78(0.58) | 6.72(0.51) | 8.83(0.65) | 8.54(0.59) |
| 50501 | Joondalup | 6.49(0.56) | 6.45(0.53) | 4.86(0.49) | 4.92(0.46) | 5.04(0.5) | 5.16(0.46) | 2.72(0.37) | 2.93(0.35) | 3.4(0.41) | 3.52(0.4) |
| 50502 | Stirling | 6.21(0.51) | 6.46(0.47) | 5.64(0.49) | 5.73(0.44) | 6.05(0.5) | 6.15(0.44) | 5.28(0.47) | 5.2(0.42) | 6.03(0.5) | 5.88(0.46) |
| 50503 | Wanneroo | 8.44(0.51) | 8.41(0.48) | 7.15(0.47) | 7.1(0.44) | 7.13(0.47) | 7.12(0.44) | 6.72(0.46) | 6.59(0.43) | 6.38(0.45) | 6.36(0.42) |
| 50601 | Armadale | 10.58(0.82) | 10.35(0.72) | 9.78(0.8) | 9.31(0.7) | 8.57(0.75) | 8.41(0.64) | 6.83(0.68) | 6.8(0.58) | 8.19(0.73) | 8.09(0.67) |
| 50602 | Belmont - Victoria Park | 6.58(0.87) | 7.39(0.78) | 7.83(0.95) | 7.79(0.8) | 6.97(0.9) | 7.36(0.75) | 5.34(0.79) | 5.68(0.67) | 6.96(0.9) | 7.34(0.8) |
| 50603 | Canning | 7.73(0.8) | 7.91(0.68) | 6.74(0.75) | 6.89(0.64) | 8.32(0.83) | 7.99(0.67) | 5.76(0.7) | 5.6(0.57) | 9.43(0.88) | 8.99(0.76) |
| 50604 | Gosnells | 8.48(0.67) | 8.68(0.62) | 7.5(0.63) | 7.59(0.58) | 6.88(0.61) | 7.21(0.55) | 6.36(0.59) | 6.45(0.54) | 10.96(0.75) | 10.73(0.71) |
| 50605 | Kalamunda | 9.56(1.09) | 8.81(0.8) | 6.09(0.89) | 6.45(0.69) | 7.62(0.99) | 7.51(0.7) | 5.68(0.86) | 5.43(0.6) | 8.59(1.04) | 7.98(0.84) |
| 50606 | Serpentine - Jarrahdale | 7.25(1.15) | 7.9(0.85) | 8.43(1.23) | 7.71(0.89) | 7.25(1.15) | 7.34(0.78) | 6.47(1.09) | 5.59(0.7) | 5.1(0.97) | 5.43(0.74) |
| 50607 | South Perth | 9.86(1.56) | 8.66(1.22) | 8.77(1.48) | 7.76(1.14) | 7.99(1.42) | 7.49(1.08) | 4.66(1.1) | 4.43(0.82) | 6.3(1.27) | 6.27(1.05) |
| 50701 | Cockburn | 8.09(0.71) | 8.1(0.61) | 5.96(0.62) | 6.26(0.54) | 7.71(0.7) | 7.55(0.57) | 4.32(0.53) | 4.59(0.44) | 5.07(0.57) | 5.45(0.53) |
| 50702 | Fremantle | 7.83(1.37) | 7.39(1.01) | 5.74(1.19) | 5.81(0.87) | 5.74(1.19) | 6.13(0.85) | 3.66(0.96) | 3.92(0.69) | 5.22(1.14) | 5.21(0.89) |
| 50703 | Kwinana | 13.49(1.31) | 12.76(1.13) | 13.2(1.3) | 12.11(1.11) | 11.19(1.21) | 10.38(1.01) | 10.26(1.16) | 9.46(0.98) | 10.26(1.16) | 9.75(1.02) |
| 50704 | Melville | 5.79(0.68) | 5.99(0.61) | 5.03(0.64) | 5.15(0.56) | 5.64(0.67) | 5.85(0.6) | 3.15(0.51) | 3.26(0.43) | 4.94(0.63) | 4.96(0.57) |
| 50705 | Rockingham | 9.74(0.66) | 9.74(0.64) | 7.94(0.61) | 7.98(0.58) | 7.48(0.59) | 7.62(0.55) | 4.77(0.48) | 5.06(0.45) | 6.28(0.54) | 6.37(0.51) |
| 50901 | Albany | 12.39(1.18) | 11.81(1.04) | 8.26(0.99) | 7.95(0.84) | 10.19(1.09) | 9.51(0.92) | 9.07(1.03) | 8.64(0.9) | 7.74(0.96) | 7.4(0.85) |
| 50902 | Wheat Belt - North | 13.4(1.31) | 12.19(0.98) | 9.28(1.11) | 9(0.86) | 10.49(1.18) | 9.62(0.82) | 6.77(0.96) | 7.77(0.76) | 5.74(0.89) | 6.91(0.78) |
| 50903 | Wheat Belt - South | 8.28(1.62) | 9.98(1.04) | 4.14(1.17) | 6.82(0.89) | 3.45(1.07) | 7.45(0.82) | 6.55(1.45) | 7.62(0.88) | 5.17(1.3) | 6.58(0.91) |
| 51001 | Kimberley | 21.39(1.69) | 21.29(1.54) | 21.56(1.69) | 21.11(1.55) | 16.81(1.54) | 16.78(1.35) | 24.62(1.78) | 24.95(1.66) | 17.49(1.57) | 17.86(1.49) |
| 51002 | East Pilbara | 13.33(1.72) | 12.78(1.3) | 13.59(1.74) | 12.28(1.3) | 12.95(1.71) | 11.57(1.17) | 16.67(1.89) | 14.68(1.47) | 10.77(1.57) | 10.17(1.24) |
| 51003 | West Pilbara | 8.7(1.21) | 9.04(1.04) | 8.89(1.22) | 8.67(1.03) | 6.51(1.06) | 7.4(0.91) | 9.09(1.24) | 9.22(1.08) | 7.78(1.15) | 7.55(0.99) |
| 51101 | Esperance | 7.62(1.83) | 9.19(1.37) | 4.31(1.4) | 6.37(1.07) | 4.78(1.48) | 7.14(1.11) | 3.81(1.32) | 6.24(1.1) | 3.81(1.32) | 5.49(1.08) |
| 51102 | Gascoyne | 9.24(2.66) | 10.92(1.93) | 6.72(2.3) | 9.1(1.73) | 6.84(2.33) | 8.79(1.61) | 12.61(3.04) | 13.34(2.3) | 7.56(2.42) | 9.01(1.88) |
| 51103 | Goldfields | 12.07(1.38) | 12.31(1.05) | 7.21(1.1) | 8.68(0.92) | 7.99(1.15) | 9.36(0.86) | 12.45(1.4) | 11.67(1.05) | 8.47(1.18) | 8.73(0.95) |
| 51104 | Mid West | 13.22(1.28) | 12.92(1.08) | 9.77(1.13) | 9.94(0.94) | 11.26(1.2) | 10.68(0.96) | 14.39(1.33) | 13.93(1.15) | 10.2(1.15) | 10.04(1) |
| ***Tasmania*** | | | | | | | | | | | |
| 60101 | Brighton | 15.02(2.16) | 14.32(1.67) | 12.45(2) | 12.27(1.5) | 15.02(2.16) | 13.42(1.51) | 13.19(2.05) | 13.13(1.6) | 9.19(1.75) | 9.06(1.35) |
| 60102 | Hobart - North East | 9.68(1.19) | 9.67(1.08) | 6.77(1.01) | 7.13(0.9) | 9.52(1.18) | 9.52(1.05) | 6.13(0.96) | 6.22(0.85) | 5.97(0.95) | 5.58(0.83) |
| 60103 | Hobart - North West | 9.75(1.15) | 10.39(1.01) | 10.64(1.19) | 10.82(1.04) | 11.26(1.22) | 11.22(1.01) | 9.91(1.16) | 10.35(1.02) | 7.35(1.01) | 7.54(0.88) |
| 60104 | Hobart - South and West | 5.2(1.06) | 6.29(0.85) | 7.01(1.21) | 7.07(0.93) | 7.94(1.29) | 7.72(0.93) | 5.69(1.11) | 5.31(0.78) | 2.94(0.8) | 3.63(0.64) |
| 60105 | Hobart Inner | 7.77(1.23) | 7.32(1) | 6.09(1.1) | 6.37(0.92) | 7.56(1.21) | 7.65(1.01) | 4.43(0.95) | 4.55(0.78) | 4.62(0.96) | 4.29(0.77) |
| 60106 | Sorell - Dodges Ferry | 12.99(2.53) | 11.25(1.6) | 10.17(2.27) | 9.35(1.41) | 9.6(2.21) | 9.85(1.39) | 9.6(2.21) | 8.17(1.33) | 3.39(1.36) | 4.87(0.97) |
| 60201 | Launceston | 8.45(0.9) | 8.55(0.85) | 8.24(0.89) | 8.33(0.82) | 7.45(0.86) | 7.66(0.79) | 7.18(0.84) | 7.32(0.79) | 5.28(0.73) | 5.24(0.67) |
| 60202 | Meander Valley - West Tamar | 7.84(1.88) | 8.52(1.16) | 8.82(1.99) | 8.4(1.22) | 6.86(1.77) | 7.89(1.04) | 8.82(1.99) | 7.65(1.14) | 4.41(1.44) | 4.8(0.88) |
| 60203 | North East | 9.97(1.56) | 10.17(1.23) | 9.41(1.51) | 9.67(1.2) | 9.21(1.51) | 9.32(1.11) | 9.39(1.53) | 9.35(1.2) | 4.58(1.09) | 5.36(0.88) |
| 60301 | Central Highlands (Tas.) | 10.88(2.57) | 11.16(1.28) | 14.29(2.89) | 11.36(1.49) | 10.2(2.5) | 10.1(1.14) | 9.52(2.42) | 9.93(1.23) | 8.16(2.26) | 7.1(1.1) |
| 60302 | Huon - Bruny Island | 12.17(2.16) | 10.82(1.64) | 11.74(2.12) | 10.61(1.62) | 6.52(1.63) | 7.82(1.27) | 10.53(2.03) | 9.46(1.57) | 10.87(2.05) | 8.66(1.57) |
| 60303 | South East Coast | 11.9(5) | 11.37(2.18) | 11.9(5) | 10.54(2.03) | 16.67(5.75) | 10.95(1.94) | 4.76(3.29) | 8.88(1.9) | 4.76(3.29) | 5.94(1.5) |
| 60401 | Burnie - Ulverstone | 10.99(1.34) | 10.77(1.19) | 10.44(1.31) | 9.97(1.16) | 10.99(1.34) | 10.43(1.15) | 9.54(1.26) | 9.35(1.14) | 4.95(0.93) | 5.24(0.83) |
| 60402 | Devonport | 5.06(0.97) | 7.08(0.93) | 5.64(1.02) | 6.97(0.92) | 7(1.13) | 7.97(0.96) | 6.42(1.08) | 7.35(0.96) | 4.09(0.87) | 4.74(0.77) |
| 60403 | West Coast | 19.37(2.86) | 13.53(1.77) | 9.42(2.11) | 9.25(1.38) | 10.99(2.26) | 9.74(1.32) | 10.47(2.22) | 9.43(1.41) | 9.95(2.17) | 7.31(1.31) |
| ***Northern Territory*** | | | | | | | | | | | |
| 70101 | Darwin City | 8.27(1.73) | 8.77(1.54) | 6.3(1.52) | 7.56(1.39) | 9.16(1.82) | 9.28(1.53) | 3.54(1.16) | 5.11(1.14) | 7.48(1.65) | 7.5(1.44) |
| 70102 | Darwin Suburbs | 11.25(1.16) | 11.59(1.04) | 11.79(1.19) | 11.81(1.08) | 9.89(1.1) | 10.26(0.97) | 12.74(1.23) | 11.81(1.1) | 9.62(1.09) | 9.45(1) |
| 70103 | Litchfield | 18.48(2.34) | 15.52(1.78) | 15.58(2.18) | 13.96(1.71) | 16.73(2.25) | 13.82(1.61) | 9.78(1.79) | 9.12(1.35) | 9.42(1.76) | 8.78(1.36) |
| 70104 | Palmerston | 13.2(1.33) | 13.28(1.24) | 13.82(1.36) | 13.64(1.25) | 9.49(1.16) | 10.02(1.05) | 8.57(1.1) | 8.95(1.01) | 7.45(1.03) | 7.76(0.97) |
| 70201 | Alice Springs | 18.48(1.71) | 18.67(1.39) | 18.68(1.72) | 18.06(1.42) | 17.77(1.69) | 16.34(1.27) | 24.56(1.9) | 23.45(1.59) | 21.25(1.81) | 19.86(1.55) |
| 70202 | Barkly | 35.29(5.8) | 32.22(3.53) | 26.47(5.35) | 27.1(3.32) | 16.18(4.47) | 19.99(2.52) | 47.06(6.05) | 44.27(4) | 27.94(5.44) | 31.12(3.93) |
| 70203 | Daly - Tiwi - West Arnhem | 28.76(2.33) | 30.83(2.15) | 32(2.41) | 33.25(2.22) | 26.02(2.28) | 26.75(1.99) | 45.16(2.58) | 47.11(2.4) | 38.56(2.51) | 39.51(2.38) |
| 70204 | East Arnhem | 28.76(2.33) | 28.95(2.14) | 32(2.41) | 31.82(2.21) | 26.02(2.28) | 25.61(2.05) | 45.16(2.58) | 44.87(2.43) | 38.56(2.51) | 38.19(2.43) |
| 70205 | Katherine | 29.69(2.55) | 27.47(2) | 30.84(2.58) | 28.16(2.06) | 22.33(2.34) | 20.79(1.72) | 35.74(2.68) | 34.22(2.21) | 29.32(2.53) | 28.06(2.16) |
| ***Australian Capital Territory*** | | | | | | | | | | | |
| 80101 | Belconnen | 12.83(0.94) | 12.29(0.84) | 12.98(0.95) | 12.45(0.86) | 11.81(0.91) | 10.94(0.79) | 7.37(0.74) | 6.88(0.62) | 9.26(0.82) | 8.73(0.74) |
| 80103 | Canberra East | NA | 18.15(2.57) | NA | 18(2.87) | NA | 13.37(1.87) | NA | 15.38(2.44) | NA | 14.89(2.85) |
| 80104 | Gungahlin | 13.03(0.92) | 12.14(0.83) | 12.66(0.91) | 11.91(0.82) | 8.24(0.75) | 8.2(0.64) | 5.78(0.64) | 5.43(0.53) | 8.91(0.78) | 8.23(0.7) |
| 80105 | North Canberra | 8.1(1.21) | 9.5(1.02) | 9.88(1.33) | 10.47(1.12) | 8.7(1.25) | 9.16(0.96) | 4.35(0.91) | 5.26(0.72) | 4.94(0.96) | 5.93(0.84) |
| 80106 | South Canberra | 12.01(1.93) | 10.62(1.28) | 18.02(2.28) | 13.8(1.57) | 14.49(2.09) | 10.84(1.25) | 7.07(1.52) | 5.65(0.87) | 8.13(1.62) | 6.75(1.06) |
| 80107 | Tuggeranong | 14.27(1.02) | 13.36(0.92) | 13.25(0.99) | 12.58(0.89) | 10.68(0.9) | 10.18(0.78) | 7.86(0.79) | 7.32(0.67) | 7.44(0.77) | 7.21(0.68) |
| 80108 | Weston Creek | 6.6(1.39) | 8.87(1.1) | 5.03(1.23) | 7.99(1.13) | 5.71(1.31) | 7.95(0.99) | 4.4(1.15) | 5.28(0.79) | 2.52(0.88) | 4.7(0.82) |
| 80109 | Woden Valley | 11.32(1.44) | 11.04(1.17) | 10.91(1.41) | 10.74(1.16) | 9.47(1.33) | 9.4(1.01) | 6.39(1.11) | 6.1(0.84) | 7.82(1.22) | 7.16(0.96) |
| 80110 | Molonglo | 9.82(2.81) | 8.55(1.29) | 11.61(3.03) | 9.2(1.45) | 7.14(2.43) | 7.91(1.14) | 2.68(1.53) | 3.68(0.67) | 7.14(2.43) | 5.07(0.99) |
| 80111 | Urriarra - Namadgi | NA | 8.65(1.19) | NA | 8.23(1.33) | NA | 7.78(1.02) | NA | 4.42(0.69) | NA | 4.91(0.92) |
